# New Alk(en)ylhydroxycyclohexanes with Tyrosinase Inhibition Potential from *Harpephyllum caffrum* Bernh. Gum Exudate

**DOI:** 10.3390/molecules27123839

**Published:** 2022-06-15

**Authors:** Olusola Bodede, Garland K. More, Roshila Moodley, Paul Steenkamp, Himansu Baijnath, Vinesh Maharaj, Gerhard Prinsloo

**Affiliations:** 1 Department of Agriculture and Animal Health, University of South Africa, Johannesburg 1709, South Africa; moregk@unisa.ac.za (G.K.M.); prinsg@unisa.ac.za (G.P.); 2 Department of Chemistry, University of Pretoria, Pretoria 0028, South Africa; vinesh.maharaj@up.ac.za; 3 Department of Chemistry, University of Manchester, Manchester M13 9PL, UK; roshila.moodley@manchester.ac.uk; 4 Research Centre for Plant Metabolomics, Department of Biochemistry, University of Johannesburg, P.O. Box 524, Auckland Park 2006, South Africa; psteenkamp@uj.ac.za; 5 School of Life Sciences, University of KwaZulu-Natal, Westville Campus, Private Bag X54001, Durban 4000, South Africa; baijnathh@ukzn.ac.za

**Keywords:** *Harpephyllum caffrum*, ^1^H NMR, cardanols, cyclohexanol, anti-tyrosinase

## Abstract

This work presents the first report on the phytochemical investigation of *Harpephyllum caffrum* Bernh. gum exudate. A known cardanol, 3-heptadec-12′-*Z*-enyl phenol (**1**) and three new alk(en)ylhydroxycyclohexanes, namely, (1*R*,3*R*)-1,3-dihydroxy-3-[heptadec-12′(*Z*)-enyl]cyclohexane (**2**) (1*S*,2*S*,3*S,*4*S,*5*R*)-1,2,3,4,5-pentahydroxy-5-[octadec-13′(*Z*)-enyl]cyclohexane (**3**) and (1*R*,2*S*,4*R*)-1,2,4-trihydroxy-4-[heptadec-12′(*Z*)-enyl]cyclohexane (**4**) were isolated from the gum. The structures of the compounds were determined by extensive 1D and 2D NMR spectroscopy and HR-ESI-MS data. The ethanolic extract of the gum was found to be the most potent tyrosinase inhibitor with IC_50_ of 11.32 µg/mL while compounds **2** and **3,** with IC_50_ values of 24.90 and 26.99 µg/mL, respectively, were found to be potential anti-tyrosinase candidates from the gum. Gum exudate may be a potential source for non-destructive harvesting of selective pharmacologically active compounds from plants. The results also provide evidence that *H. caffrum* gum may find application in cosmetics as a potential anti-tyrosinase agent.

## 1. Introduction

*Harpephyllum* Bernh. is a monotypic genus of the family Anacardiaceae with *Harpephyllum caffrum* Bernh. as the only recognised species. Anacardiaceae, also known as the cashew family, is characterised by gum/resin secretion [1,2]. The ethnomedicinal uses of *H. caffrum* include the treatment of skin diseases such as acne and eczema, control and management of infant convulsions and epilepsy and for blood purification [3,4]. Phytochemical studies of *H. caffrum* have been documented for its leaves [5,6], edible fruits and stem bark [7]. These studies revealed the presence of polyphenolic compounds and their glycosides, lupane-type triterpenoids, alkyl coumarates and cardanols with saturated and unsaturated alkyl chains. The cardanols have similar alkyl chains with alkenyl cyclohexanols but differ in the ring type, the latter being non-aromatic [5,6,7]. However, no information is available on the natural gum exudate produced by this species.

Plant gum exudates are often harvested for their pharmaceutical [8], food [9] and cosmetic applications [10,11]. They serve as binders due to their rich structurally stable carbohydrate and protein compositions [12] and are preferred over their synthetic polymer counterparts because of their biodegradability, non-toxic nature and economic importance [8]. Although the chemistry of gums has reportedly been characterised by several polysaccharides [12,13], terpenoids have been found occurring in large amounts for some gums [14,15], whereas alkenyl cyclohexanols have not been reported from plant gums. We herein report on the phytochemistry of *H. caffrum* gum exudate using chromatographic separations and spectrometric techniques. A preliminary study on the anti-tyrosinase potential of the isolated compounds is also evaluated.

## 2. Results and Discussion

### 2.1. Chemistry of Isolated Compounds

**Figure 1:**
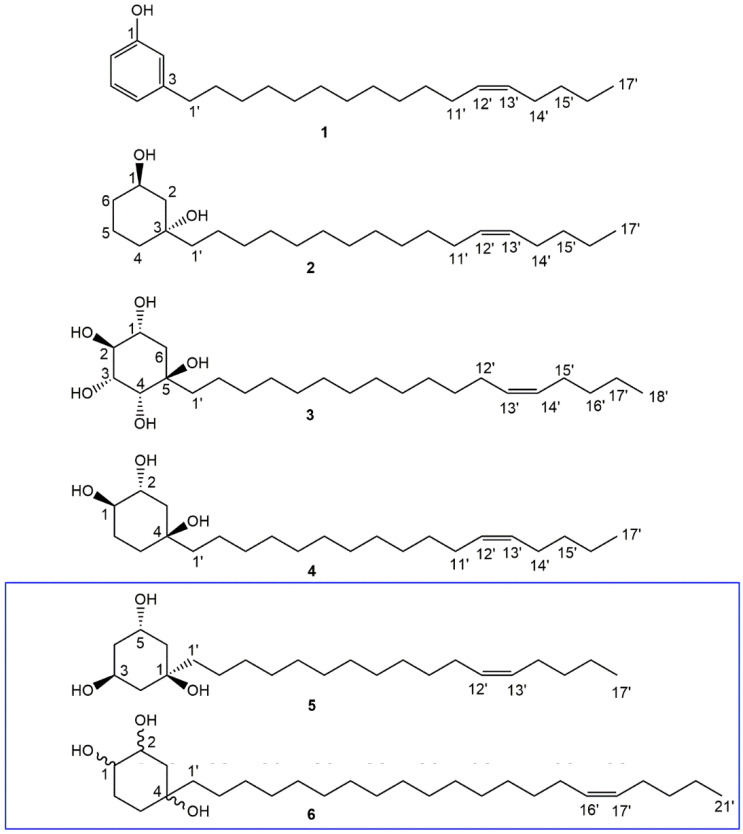
Structures of compounds isolated from *H. caffrum* gum (compounds **1**–**4**) and two (**5** and **6**) closely related compounds to **4**.

Compound **1** (61 mg) was isolated as a yellow oil. The ^1^H and ^13^C NMR data (Table 1 and Table 2) of **1** were consistent with those reported by Okoth et al. (2016) for the cardanol, 3-heptadec-12′-Z-enyl phenol [16]. Its molecular formula was obtained as C_23_H_38_O based on HR-ESI-MS (*m*/*z* 329.2823 [M-H]^−^; calcd for C_23_H_37_O, 329.2844). This single mass analysis further justified the assignment of alkenyl chain length of the proposed cardanol. The structures of **1** and other compounds isolated from *H. caffrum* gum are presented in Figure 1.

**Figure 2:**
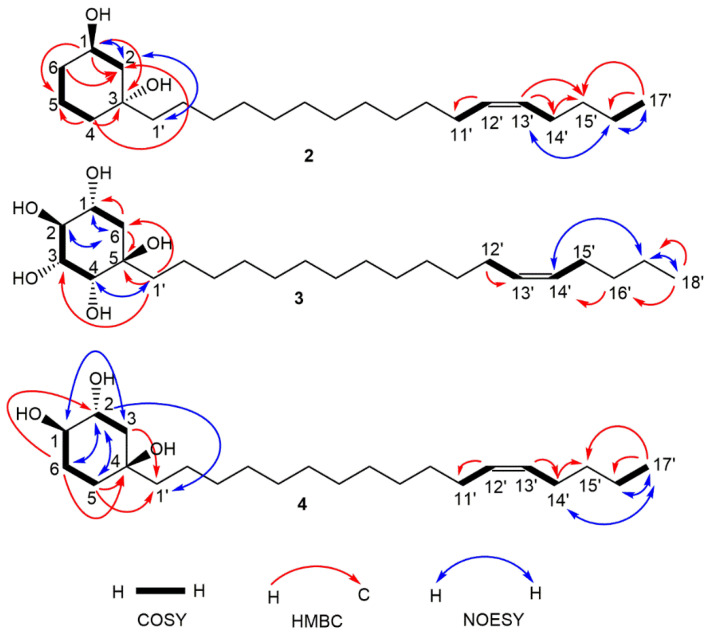
Major correlations, COSY (thick black line), HMBC (red arrow) and NOESY (blue arrow), observed for compounds **2**, **3** and **4**.

**Figure 3:**
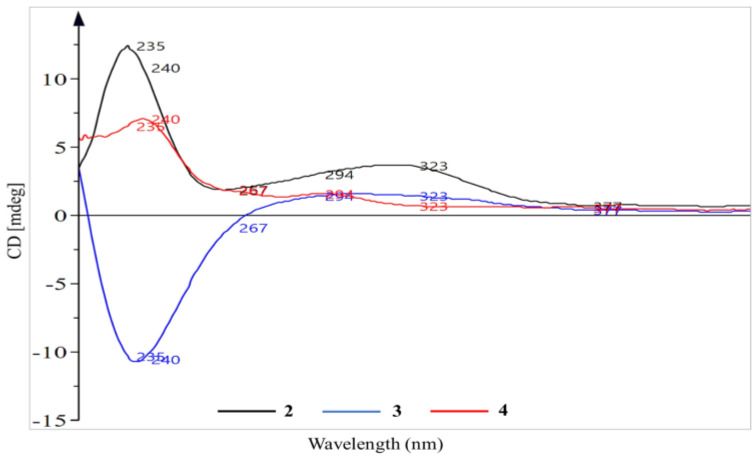
ECD spectra of compounds **2**, **3** and **4**.

Similarly to **1**, compound **2** (0.54 g) was isolated as a yellow oil, having a molecular formula of C_23_H_44_O_2_ obtained from HR-ESI-MS (*m*/*z* 375.3225 [M+Na]^+^; calcd for C_23_H_44_O_2_Na, 375.3239). The MS report also gave a DBE of **2**, suggesting a cyclohexane rather than the benzene ring system in **1**. The ^1^H NMR data (Table 1) of **2** displayed a disubstituted cyclohexane ring system with the oxygenated methine proton resonance at δ_H_ 4.07 (brs, H-1). In addition, the non-equivalent pairs of proton resonances at δ_H_ 1.95 (m, H-5a) and δ_H_ 1.43 (m, H-5b), δ_H_ 1.83 (m, H-2a) and δ_H_ 1.48 (H-2b), δ_H_ 1.60 (m, H-6a) and δ_H_ 1.35 (m, H-6b) as well as δ_H_ 1.37 (bd, J = 1.7 Hz, H-4) were assigned to complete the resonances of the cyclohexane ring. The proton resonances corresponded to δ_C_ 67.8 (C-1), 16.0 (C-5), 41.2 (C-2), 36.9 (C-6) and 43.7 (C-4) while the only quaternary carbon resonated at δ_C_ 72.9 (C-3) in the ^13^C NMR spectrum. These assignments were supported by COSY correlations between H-1 and H-2, H-4 and H-5, H-5 and H-6, in addition to HMBC the correlations of H-1 with C-2, C-3 and C-5, H-4 with C-2, C-3 and C-5 and H-6 with C-2. NOESY correlations were only observed between H-1 and H-2, and H-2 and H-1′ on the cyclohexane ring while H-16′ had NOESY correlations with both H-13′ and H-17′, observed on the alkenyl chain. The placement of double bond was further justified by HMBC correlations of H-13′ with C-14′ and C-15′ in addition to those of H-17′ with C-16′ and C-15′. The olefinic protons with a characteristic multiplet at δ_H_ 5.32 and resonances of C-11′ and C-14′ (adjacent to the double bond) observed at δ_C_ 27.2 and 26.9, respectively, indicated a cis mono-unsaturated alkyl chain [16,17]. All COSY, HMBC and NOESY correlations are shown in Figure 2. The absolute configuration of **2** was deduced from the spectrum of the electronic circular dichroism (ECD) (Figure 3), which revealed a high-amplitude positive cotton effect in the regions 230–250 and 295–325 nm. Compound **2** was therefore named (1R,3R)-1,3-dihydroxy-3-[heptadec-12′(Z)-enyl]cyclohexane.

Compound **3** (1.40 g) obtained as a colourless oil differs significantly from **2** and **4** by the length of the hydrocarbon side chain and its polyhydroxylated nature of the cyclohexane ring. This was revealed by the HR-ESI-MS analysis (*m/z* 413.3285 [M-H]^−^; calcd for C_24_H_45_O_5_, 413.3267) which afforded molecular formula of C_24_H_46_O_5_. In the ^1^H NMR data of **3** (Table 1), four oxygenated methine protons were observed at δ_H_ 3.91 (m, H-1), δ_H_ 4.01 (bs, H-2), δ_H_ 4.47 (bs, H-3) and δ_H_ 3.41 (bd, *J* = 9.8 Hz, H-4). A pair of non-equivalent proton resonances at δ_H_ 1.93 (m, H-6a) and δ_H_ 1.22 (m, H-6b) were assigned to the only hydroxy free site in the cyclohexane ring. The corresponding cyclohexane carbons were observed at 67.6 (C-1), 66.8 (C-2), 65.9 (C-3), 72.7 (C-4), 73.6 (C-5) and 45.9 (C-6). COSY correlations were between H-1 and H-6, H-2 and H-3 and H-3 and H-4 while NOESY correlations were observed between H-2 and H-6 and H-4 and H-1′ which supported the position of the alkenyl chain on the ring. This was further justified by HMBC correlations of H-1′ to C-4, C-5 and C-6. The various NOESY and HMBC correlations between positions 12′ and 18′ were similar to those observed for **2**; thus, the location of the double bond was assigned. The double bond was also assigned cis, similar to **2**. Unlike **2**, the ECD spectrum of **3** (Figure 3) showed a high-amplitude positive cotton effect in the regions 230–250 and a weak one around 295–325 nm. Compound **3** was therefore named (1*S*,2*S*,3*S,*4*S,*5*R*)-1,2,3,4,5-pentahydroxy-5-[octadec-13′(*Z*)-enyl]cyclohexane.

**Figure 4:**
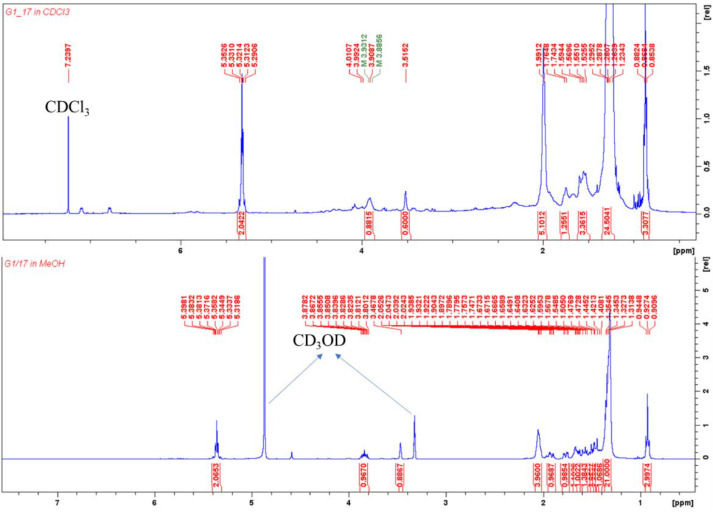
^1^H NMR spectra of compound **4** (comparing the run in CDCl_3_ with CD_3_OD).

Compound **4** (1.04 g) was obtained as a brown oil. In comparison to compound **2** (with one oxygenated methine proton), the ^1^H NMR spectrum (Table 1) displayed a cyclohexane ring consisting of two deshielded oxygenated methine protons at δ_H_ 3.46 (bs, H-1) and δ_H_ 3.86 (m, H-2), whose multiplicities suggested they are equatorial and axial, respectively [18]. The three methylene proton pairs in the cyclohexane ring were observed at δ_H_ 1.89–1.93 (1H, dt, *J* = 13.98, 3.0 Hz, H-5a) and 1.62–1.64 (1H, m, H-5b), 1.78 (1H, dd, *J* = 12.90, 4.03 Hz, H-6a) and 1.47 (1H, m, H-6b) and 1.45–1.50 (2H, m, H-3a&b). Their positions on the cyclohexane ring were ascertained by COSY correlations between H-1 and H-5a and H-6a and between H-2 and H-3a&b which were supported by HMBC correlations: H-6a to C-2 and C-4; H-5a to C-1′; H-5b to C-4 and C-1′; and H-3 to C-1′. These correlations (with C-1′) also justify the placement of the alkenyl chain at position C-4 on the ring. However, resonances of the alkenyl side chain were similar to those of **2** and **3**, with the olefinic protons resonating at δ_H_ 5.36 while C-11′ and C-14′ (adjacent to the double bond) were observed at δ_C_ 28.1 and 27.9, respectively, for a cis alkene. The location of the double bond was determined based on HMBC correlations between olefinic protons and the allylic carbons (C-11′ and 14′), H-14′ and C-15′ and H-17′ and C-14′/C-15′. HR-ESI-MS at m/z 369.3301 [M+H]^+^ (calcd, 369.3369). The ECD spectrum of **4** was similar to that of **2** (Figure 3), which revealed a positive cotton effect in the regions 230–250 and 295–325 nm. Compound **4** was thus identified as (1*R*,2*S*,4*R*)-1,2,4-trihydroxy-4-[heptadec-12′(*Z*)-enyl]cyclohexane. Due to the structural similarity (especially on the substituted cyclohexane moiety) between compound **4** and the 1-alkenyl-1,3,5-trihydroxycyclohexane (**5**), reported by Laurent et al. (2003) [18], and 1,2,4-trihydroxy-4-[16′(Z)-heneicosenyl]cyclohexane (**6**), reported by Oktoth et al. (2016) [16], a detail comparison list is provided in Table 1. Compound **4** had better solubility and good peak resolution in CD_3_OD compared to CDCl_3_. However, the ^1^H NMR was run in both solvents (Figure 4) for accurate comparison with **6** which was run in CDCl_3_. The oxygenated methine protons of **4** had chemical shifts that were more upfield (δ_H_ 3.51 and 3.90) in comparison to **5** (δ_H_ 4.31 and 4.35). However, the same methine protons had similar values with those of **6**, justifying their similar positions, while the differences in their multiplicities suggested a difference in their configuration. Compound **6** further differed from **4** because of its longer alkenyl chain length. All spectral data (Figures S1 to S36) for compounds 1 to 4 are available in the Supplementary Material.

In previous studies, phenolic lipids such as cardanols, cardols and anacardic acids have been identified from different species of Anacardiaceae such as *Anacardium occidentale* [19], *Mangifera indica* [20] and *H. caffrum* [7], making them chemotaxonomically significant for this “cashew family” [21] and highlighting that some bioactive compounds believed to be localised in specific plant parts can be found in the “plant waste” gum. Contrastingly, alkyl and alk(en)ylhydroxycyclohexanes have not gained popularity in Anacardiaceae. Roumy et al. (2009) initially reported four cyclic alkyl polyol derivatives from *Tapirira guianensis* [17] while Okoth et al. (2016) reported an alk(en)ylhydroxycyclohexane alongside close cyclohexanone derivatives and some phenolic lipids [16]. The present study suggests that the alk(en)ylhydroxycyclohexane may be part of yet-to-be-identified phytochemicals present in the *H. caffrum* tree. Further studies involving other species of Anacardiaceae will be required to establish if this rare class of compounds also has chemotaxonomic importance to the family.

### 2.2. Tyrosinase Inhibitory Activity

The anti-tyrosinase inhibitory activity of the isolated compounds (**1**–**4**) and *Harpephyllum caffrum* gum-ethanolic extract HCG-EtOH was evaluated using _L_-tyrosine as substrate. The test solutions for each compound and extract were prepared with concentrations varying from 1.56 to 200 μg/mL and results are presented as half maximum inhibitory concentration (IC_50_) shown in Table 3. The ethanolic extract, HCG-EtOH, was identified as potent tyrosinase inhibitors with IC_50_ value (11.32 µg/mL) comparable with that of the control, arbutin (9.85 µg/mL) although lower than kojic acid (4.34 µg/mL). Compounds **2** and **3** showed good inhibition with IC_50_ values of 24.90 and 26.99 µg/mL, respectively, while **1** and **4** were moderately active with IC_50_ values of 41.77 and 34.90 µg/mL, respectively. The results suggest that HCG-EtOH’s potent anti-tyrosinase activity may be due to the synergistic effects of its constituents, of which **2** and **3** possibly play a significant role in the enzyme–substrate interactions. However, this proposed synergy warrants further investigation. We opined that the basic cyclohexanol moiety of **2**–**4** (IC_50_ between 24.90 and 34.90 µg/mL) could be a better tyrosinase inhibition pharmacophore than phenolic in **1** (IC_50_; 41.77 µg/mL), since all compounds had similar alkenyl side chains. Tyrosinase inhibitory potential of alk(en)ylhydroxycyclohexanes has not been previously reported. Yu et al. (2016) reported earlier that cardanols exhibited their anti-tyrosinase activity by altering tyrosinase conformation and significantly decreasing the steady state rate of tyrosinase diphenolase activity [22]. A previous study showed that the stem bark of *H. caffrum* could be considered as an anti-tyrosinase agent with an IC_50_ of 40 µg/mL observed for its ethanolic extract [23]. Therefore, it is most likely that gum exudate from *H. caffrum* is a better anti-tyrosinase agent compared to the stem bark as revealed in the current study (IC_50_ of HCG-EtOH, 11.32 µg/mL).

## 3. Materials and Methods

### 3.1. General Experimental Procedures

The infrared (IR) spectra were obtained on a Perkin Elmer Spectrum 100 Fourier transform infrared spectrophotometer (FT-IR) with universal attenuated total reflectance (ATR) sampling accessory. Electronic Circular Dichroism (ECD) data were acquired using an Applied Photophysics Chirascan spectrometer (Applied Photophysics Ltd., Leatherhead, UK). ^1^H, ^13^C and 2D nuclear magnetic resonance (NMR) spectra were recorded using deuterated methanol (CD_3_OD) at room temperature on a Bruker Avance^III^ 400 MHz spectrometer. The high-resolution electrospray ionization mass spectra (HR-ESI-MS) were obtained on a Waters SYNAPT G1 High-Definition Mass Spectrometer (Waters, MA, USA). All column chromatography (CC) was carried out using Merck silica gel 60 (0.040–0.063 mm) while Merck 20 cm × 20 cm silica gel 60 F_254_ aluminium sheets were used for thin-layer chromatography (TLC). The TLC plates were analysed under UV (254 and 366 nm) before further visualisation by spraying with 10% sulfuric acid in methanol (MeOH) solution followed by heating. Analytical grade solvents and other chemicals used were supplied by either Merck (Darmstadt, Germany) or Sigma (St. Louis, MO, USA) chemical companies.

### 3.2. Sample Collection and Identification

Gum was collected from *H. caffrum* bark growing in Reservoir Hills, Durban. The *H. caffrum* tree has been identified and a voucher specimen (R. Moodley 2) was deposited in the Ward Herbarium, School of Life Sciences, University of KwaZulu-Natal, Westville, Durban, South Africa [7].

### 3.3. Extraction and Purification

Four hundred grams of *H. caffrum* gum (HCG) was crushed (using a mortar and pestle) without further drying. The crushed gum was then extracted with ethanol (EtOH) and the resulting extract was concentrated using a rotary evaporator to obtain HCG crude EtOH extract (HCG-EtOH). The HCG-EtOH (2 g) was subjected to CC using varying ratios of hexane:EtOAc (from 1:0 to 0:1, *v*/*v*) and EtOAc:MeOH (from 1:0 to 0.9:0.1, *v*/*v*) as mobile phases. A total of 30 aliquots were collected from which aliquots 5 and 11 yielded compounds **1** and **2**, respectively. The remaining aliquots were combined based on TLC profiles to give A (aliquots 13–16), B (17–20) and C (21–30). Fraction C (520 mg) was re-chromatographed using a gradient elution similar to the one described for the CC of HCG-EtOH, resulting in compounds **3** and **4**.

### 3.4. Tyrosinase Inhibitory Assay

Tyrosinase inhibitory activity was determined spectrophotometrically with slight modifications [24]. Briefly, tested samples were dissolved in DMSO to a final concentration of 20 mg/mL. In a 24-well plate, 30 μL of the samples was added to 970 μL of potassium phosphate buffer (0.1 M, pH 6.5) and serially diluted two-fold. The mixture (70 μL) was transferred to a 96-well plate containing 30 μL of potassium phosphate buffer (0.1 M, pH 6.5) to obtain increasing concentration range of (1.56–200 μg/mL). Then, 30 μL of mushroom tyrosinase solution (333 Units/mL in phosphate buffer, pH 6.5) was added to each mixture, which was then incubated at 25 °C for 5 min. Thereafter, 110 µL of 2 mM L-tyrosine was added in all wells of a 96-well microtiter plate and further incubated for 30 min at 25 °C. Arbutin and kojic acid were included as positive controls tested at concertation range (1.56–200 μg/mL) and 1% DMSO was included as a negative control. The absorbance of the mixture was read at 492 nm using an ELISA microplate reader (VarioSkan Flash, Thermo Fisher Scientific, Vantaa, Finland). All experiments were performed in triplicates. The percent inhibition of tyrosinase was calculated as follows:

Tyrosinase inhibitory (%)=(Ao−A1Ao) × 100

where *A*_0_ is the absorbance of the negative control with the enzyme without test samples, *A*_1_ is the absorbance of the test samples and enzyme.

### 3.5. Statistical Analysis

Data were expressed as mean ± SD from three independent experiments. The IC_50_ values, corresponding to the concentration required to inhibit 50% of tyrosinase activity, were calculated from a sigmoidal dose–response of a non-linear regression and R-square values representing the best fit of the model were determined by Pearson nonlinear regression using the GraphPad Prism 5 software (GraphPad Software, San Diego, CA, USA). A one-way ANOVA was performed and differences between means were separated using the Tukey multiple comparison test at *p* < 0.05. Experiments were performed in triplicate.

## 4. Conclusions

Three new alkenyl cyclohexanols of the order, di, tri and pent-ol, were isolated from the gum of *Harpephyllum caffrum*, alongside a known cardanol. The alkenyl side chain of compound **3** differs from other compounds with an additional methylene unit which was deduced from HR-ESI-MS report. All compounds show moderate inhibition of tyrosinase while the ethanolic extract of the gum displayed potent activity, comparable to arbutin. Gum exudate of *H. caffrum* may be a potential anti-tyrosinase candidate with cosmetic applications. However, further studies which evaluate the toxicity of the isolates and extracts are required to establish this preliminary report.

## Figures and Tables

**Table 1:** ^1^H NMR [400 MHz, δ_H_, mult. (*J* in Hz)] spectral data for compounds **1–4** isolated from *H. caffrum* gum and **5** and **6**, structurally similar to **4**.

Position		1	2	3	4		* 5	** 6
					CDCl_3_	CD_3_OD	CDCl_3_	
1	1		4.07, bs	3.91, m	3.51, bs	3.46, bs		3.41, dd (10.5, 4.6)
2	2	6.63, s	1.83, m & 1.48, m	4.01, bs	3.90, m	3.85, m	1.91, bd (14.4) & 1.45, m	4.01, bs
3	3			4.47, bs	1.46–1.57, m	1.45–1.50, m	4.31, bs	1.96–1.98, m & 1.45, m
4	4	6.74, d (7.5)	1.38, bd (1.86)	3.41, bd (9.8)			2.29, bd (12.5) & 1.40, m	
5	5	7.11, t (7.9)	1.95, m & 1.43, m		1.89–1.97, m & 1.66, m	1.89–1.93, dt (13.9, 3.0) & 1.62–1.64, m	4.35, tt (11.4, 4.2)	1.86, m & 1.62, m
6	6	6.61, dd (8.4, 2.3)	1.60, m & 1.35, m	1.93, m & 1.22, m	1.74, bd (12.0)	1.78, dd (12.9, 4.0)	2.11, bd (12.5) & 1.32, m	1.53, m
1′	1′	2.54, t (7.5)	1.75, m & 1.44, m	1.42, m		1.66–1.67, m & 1.54–1.59, m	1.48, m & 1.38, m	1.84, m & 1.48, m
2′–10′	2′–14′	1.24–1.57, m	1.23–1.32, m	1.10–1.26, m & 1.93		1.29–1.36, m	1.30–1.50, m	1.20–1.30, m
11′	15′	1.99, m	1.96–1.99, m	1.23–1.44, m	1.96–2.01, m	2.02–2.05, m	2.03, m	1.98–2.02, m
12′	16′	5.34, m	5.31, m	1.96–1.99, m	5.32, m	5.36, m	5.37, m	5.32, t (5.0)
13′	17′	5.34, m	5.31, m	5.32, m	5.32, m	5.36, m	5.37, m	5.32, t (5.0)
14′	18′	1.99, m	1.96–1.99, m	5.32, m	1.96–2.01, m	2.02–2.05, m	2.03, m	1.98–2.02, m
15′	19′	1.24–1.30, m	1.28–1.31, m	1.96–1.99, m		1.29–1.36, m	1.30–1.50, m	1.20–1.30, m
16′	20′	1.24–1.30, m	1.28–1.31, m	1.31, m		1.30 1.42–1.44, m	1.30–1.50, m	1.20–1.30, m
17′	21′	0.88, t (6.8)	0.87, t (6.8)	1.28–1.31, m	0.89, t (5.7)	0.92, t (6.9)	0.91 (6.6)	0.86, t (6.8)
18′				0.86, t (6.8)				

Compounds **1**–**3** were recorded in CDCl_3_ while **4** was recorded in both CDCl_3_ and CD_3_OD; * Laurent et al. 2003 [18]; ** Okoth et al. 2016 [16].

**Table 2:** ^13^C NMR (400 MHz, δ_C_, Type) spectral data for compounds **1–4**.

Position	1	2	4	Position	3
1	155.4, C	67.8, CH	70.9, CH	1	67.6, CH
2	115.3, CH	41.2, CH_2_	67.7, CH	2	66.8, CH
3	144.9, C	72.9, C	40.6, CH_2_	3	65.9, CH
4	120.9, CH	43.7, CH_2_	76.1, C	4	72.7, CH
5	129.3, CH	16.0	28.3, CH_2_	5	73.6, C
6	112.4, CH	36.9	41.9, CH_2_	6	45.9, CH_2_
1′	35.8, CH_2_	32.8, CH_2_	29.8, CH_2_	1′	44.5, CH_2_
2′	31.3, CH_2_	22.8, CH_2_	23.3, CH_2_	2′	35.3, CH_2_
11′	27.2, CH_2_	27.2, CH_2_	28.1, CH_2_	12′	27.2, CH_2_
12′	129.8, CH	129.8, CH	130.8, CH	13′	129.8, CH
13′	129.9, CH	129.9, CH	130.8, CH	14′	129.9, CH
14′	26.9, CH_2_	26.9, CH_2_	27.9, CH_2_	15′	26.9, CH_2_
15′	31.9, CH_2_	31.9, CH_2_	33.1, CH_2_	16′	31.9, CH_2_
16′	22.3, CH_2_	22.3, CH_2_	23.1, CH_2_	17′	22.3, CH_2_
17′	14.0, CH_3_	13.9, CH_3_	14.3, CH_3_	18′	13.9, CH_3_

Compounds **1**–**3** were recorded in CDCl_3_ while **4** was recorded in CD_3_OD.

**Table 3:** Tyrosinase inhibitory activity of different extracts and compounds from *Harpephyllum caffrum* (HCG-EtOH) compared with the positive control: arbutin and kojic acid.

Test Samples	Anti-Tyrosinase IC50 ± SD (µg/mL)	Correlation Coefficient (R2)
HCG-EtOH	^b^ 11.32 ± 0.80	0.9892
Compound **1**	^e^ 41.77 ± 0.62	0.9813
Compound **2**	^c^ 24.90 ± 1.10	0.9470
Compound **3**	^c^ 26.99 ± 1.30	0.9659
Compound **4**	^d^ 34.90 ± 0.73	0.9731
Arbutin	^b^ 9.85 ± 0.42	0.9577
Kojic acid	^a^ 4.34 ± 0.37	0.9969

IC_50_ values are presented as mean ± SD and the lower the IC_50_ value, the better the anti-tyrosinase effect. IC_50_ values with the same superscript letter are not significantly different.

## Data Availability

Not applicable.

## Supplementary Materials

The following supporting information can be downloaded at: https://www.mdpi.com/article/10.3390/molecules27123839/s1, Figure S1 to S36 containing 1D and 2D NMR spectra, FT-IR, ECD spectra and high-resolution mass spectra of compounds **1**–**4**.

## Sample Availability

Samples of the compounds (**1**–**4**) are available from the authors.
