# Supplementary material for: New Alk(en)ylhydroxycyclohexanes with Tyrosinase Inhibition Potential from Harpephyllum caffrum Bernh. Gum Exudate
Source: Molecules. 2022 Jun 15;27(12):3839. doi: 10.3390/molecules27123839 (PMC9229927; doi:10.3390/molecules27123839)
Supplement: Supplementary file 1 [file molecules-27-03839-s001.zip › molecules-1760167-supplementary.pdf]

SUPPLEMENTARY MATERIAL

New alk(en)ylhydroxycyclohexanes with tyrosinase inhibition potential from *Harpephyllum caffrum* Bernh. gum exudate

Olusola Bodede[1,5*], Garland K. More[1], Roshila Moodley[2], Paul Steenkamp[3], Himansu Baijnath[4], Vinesh Maharaj[5], Gerhard Prinsloo[1]

[1]Department of Agriculture and Animal Health, University of South Africa, Florida, Johannesburg, 1709 South Africa

[2]Department of Chemistry, University of Manchester, United Kingdom

[3]Research Centre for Plant Metabolomics, Department of Biochemistry, University of Johannesburg, P.O. Box 524, Auckland Park 2006, South Africa

[4]School of Life Sciences, University of KwaZulu-Natal, Westville Campus. Private Bag X54001, Durban, 4000 South Africa

[5]Department of Chemistry, University of Pretoria, Hatfield 0028, South Africa
*Correspondence: olusolabodede@gmail.com; ORCID 0000-0002-7265-1377

The NMR, FTIR and ECD spectral and HR-ESI-MS data and for compounds **1** to **4** are presented in Figure S1 to S20

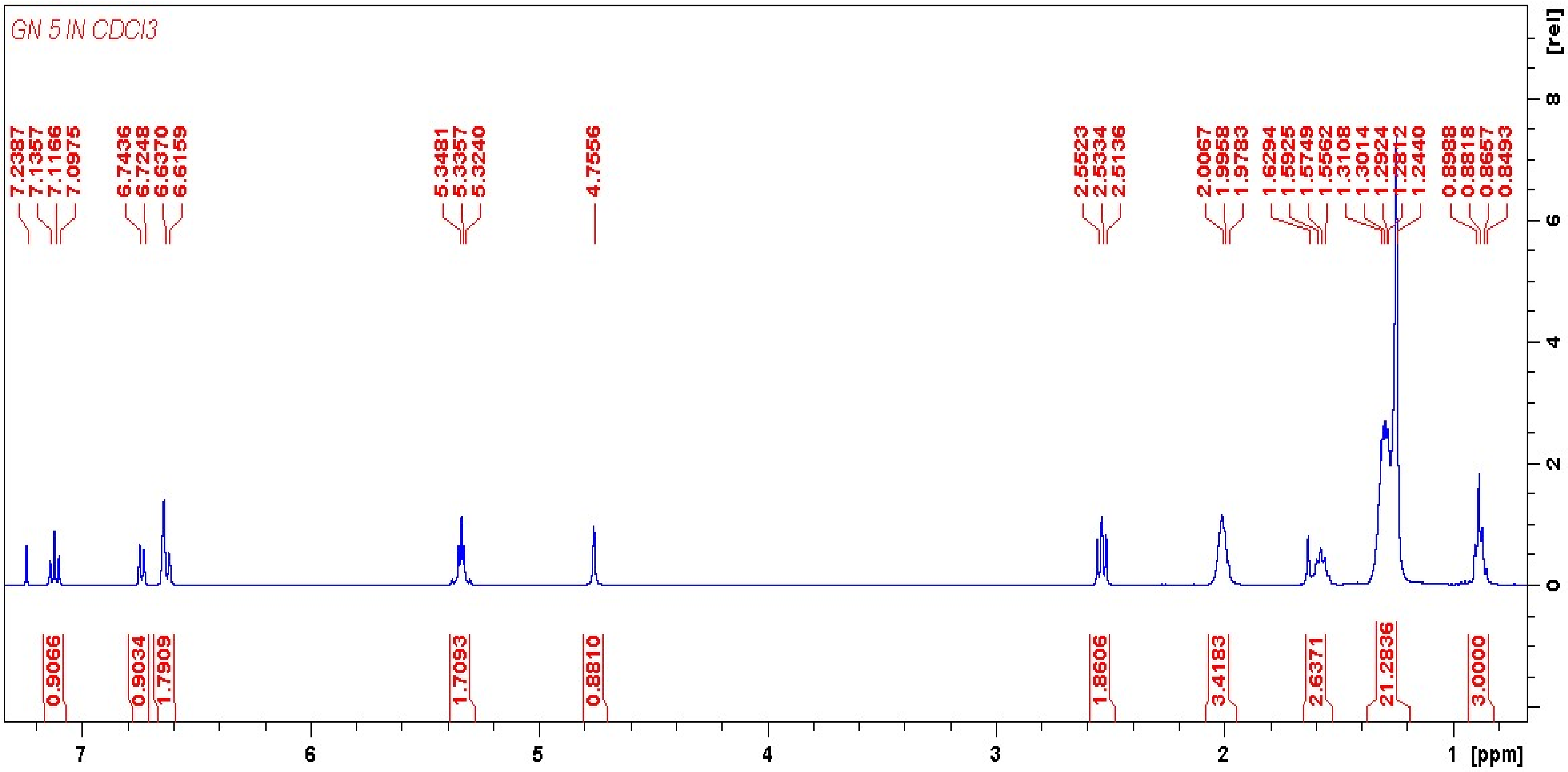

Figure S1. ^{1}H-NMR spectrum for 3-heptadec-12′-*Z*-enyl phenol (**1**)

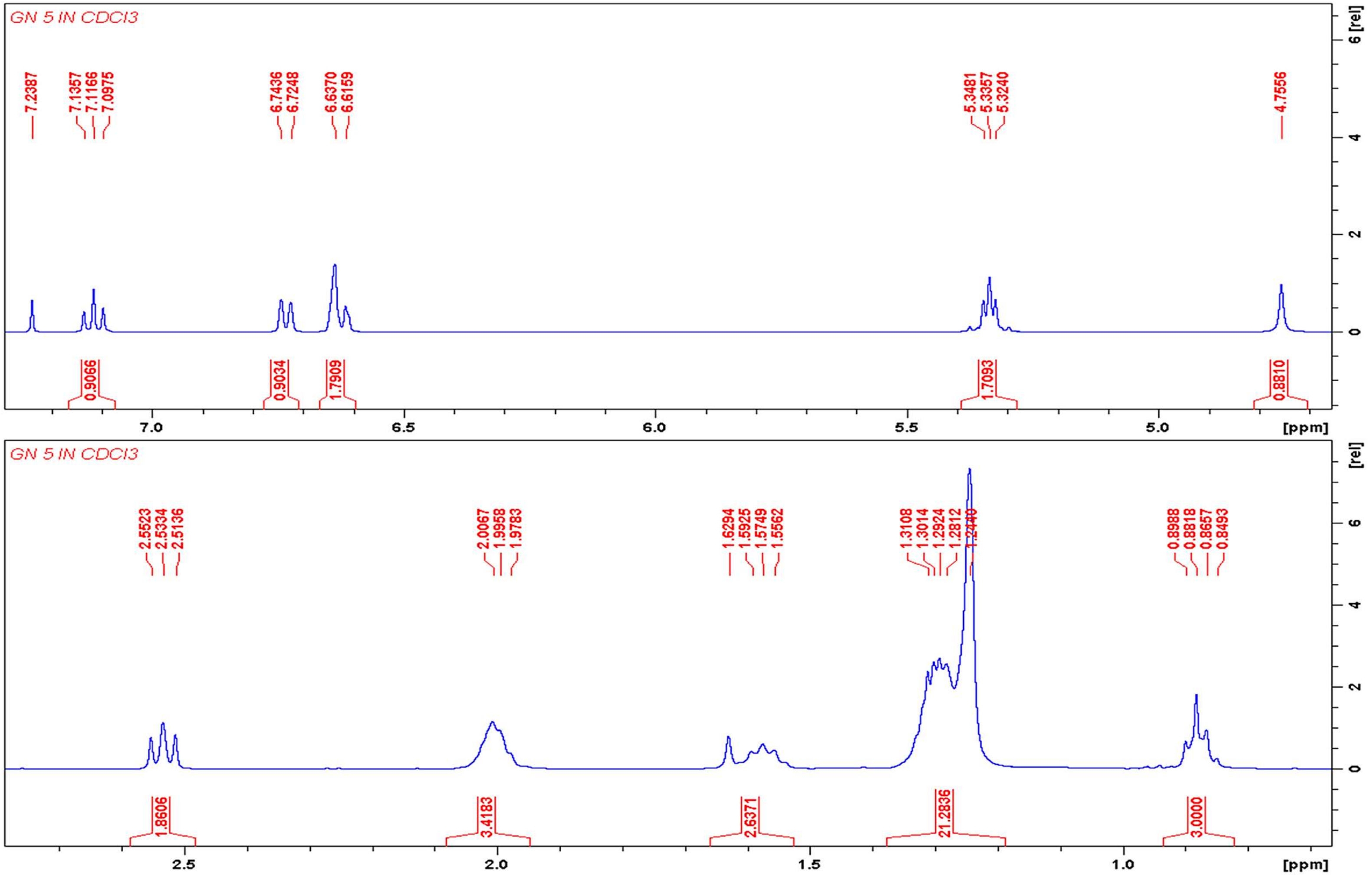

Figure S2. Expanded ^{1}H-NMR spectrum for 3-heptadec-12′-*Z*-enyl phenol (**1**)

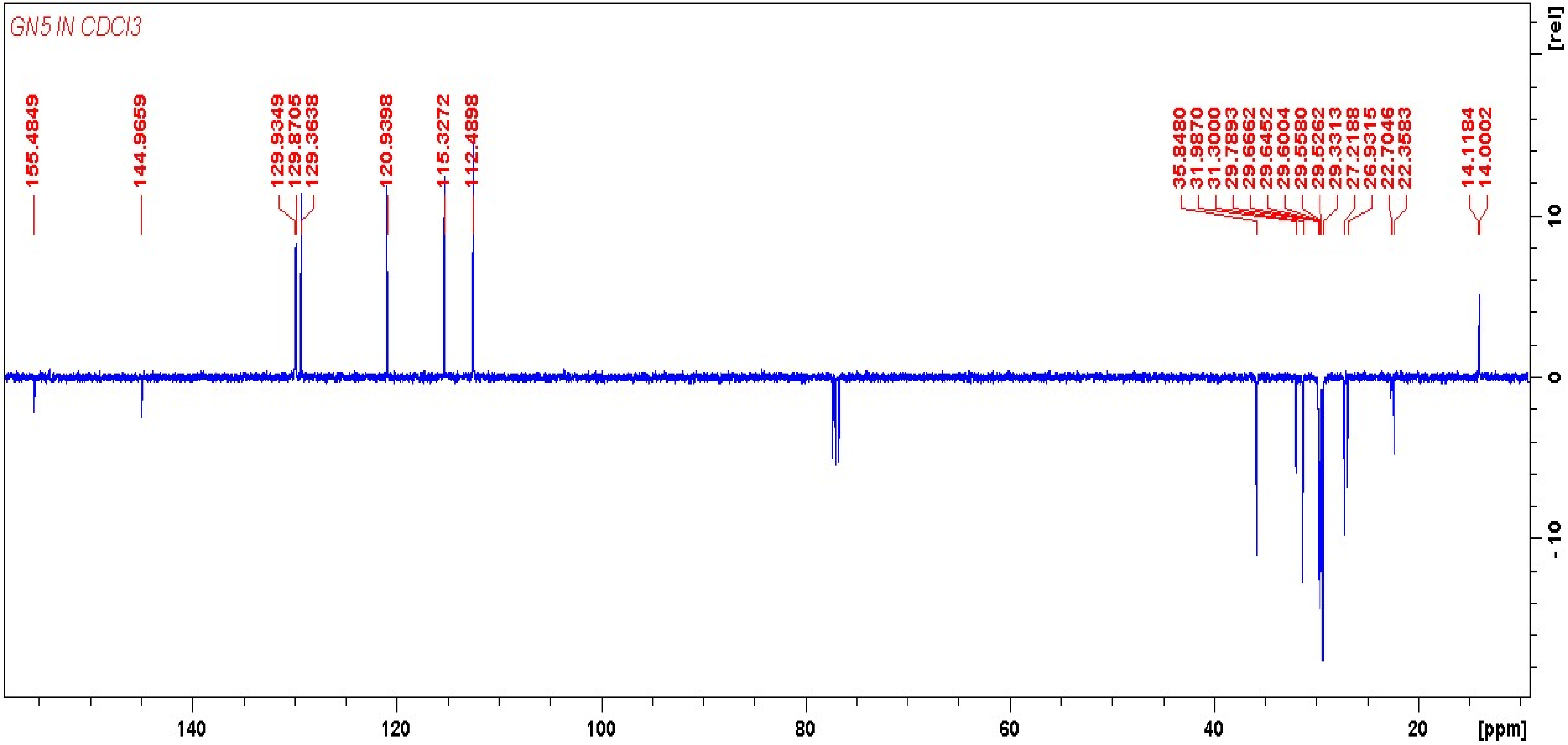

Figure S3. APT spectrum for 3-heptadec-12′-*Z*-enyl phenol (**1**)

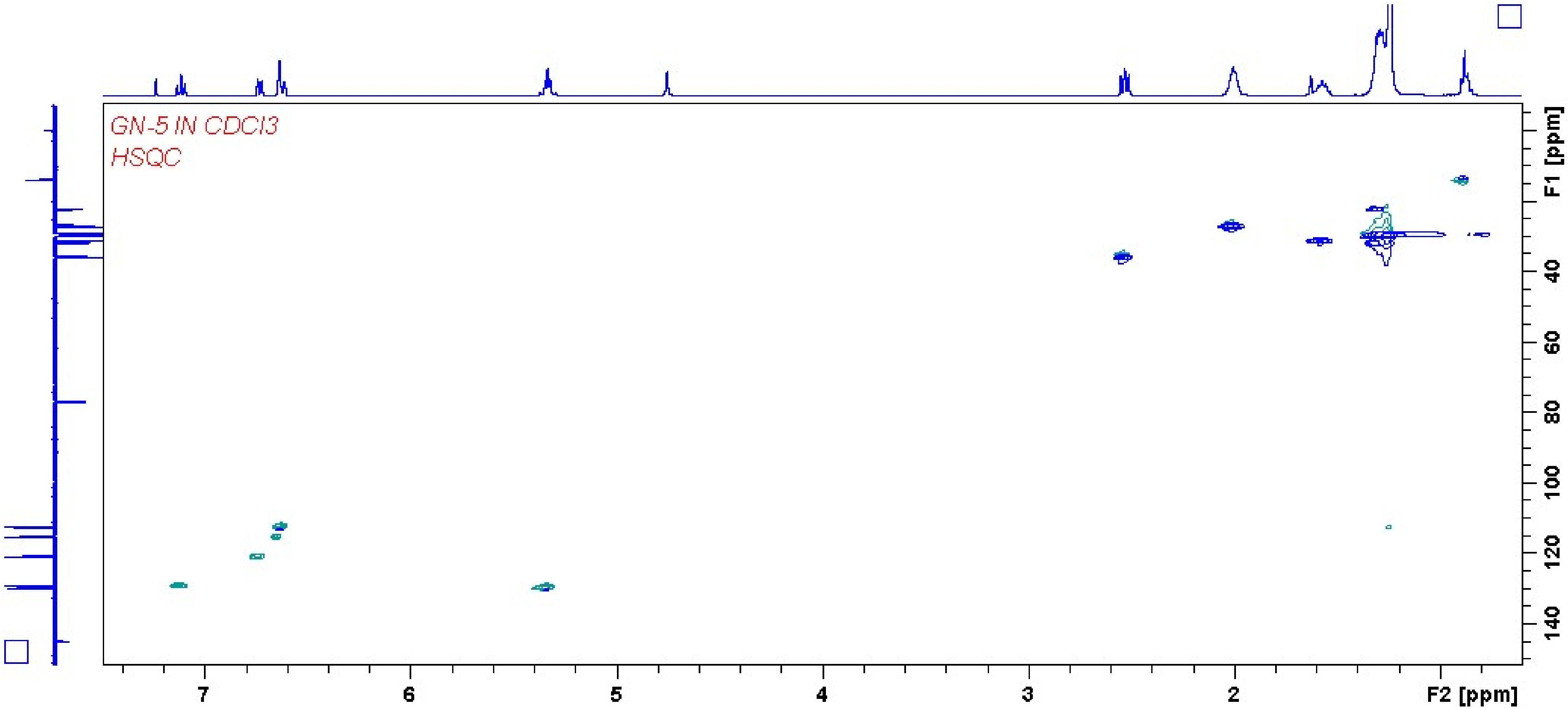

Figure S4. HSQC spectrum for 3-heptadec-12′-Z-enyl phenol (**1**)

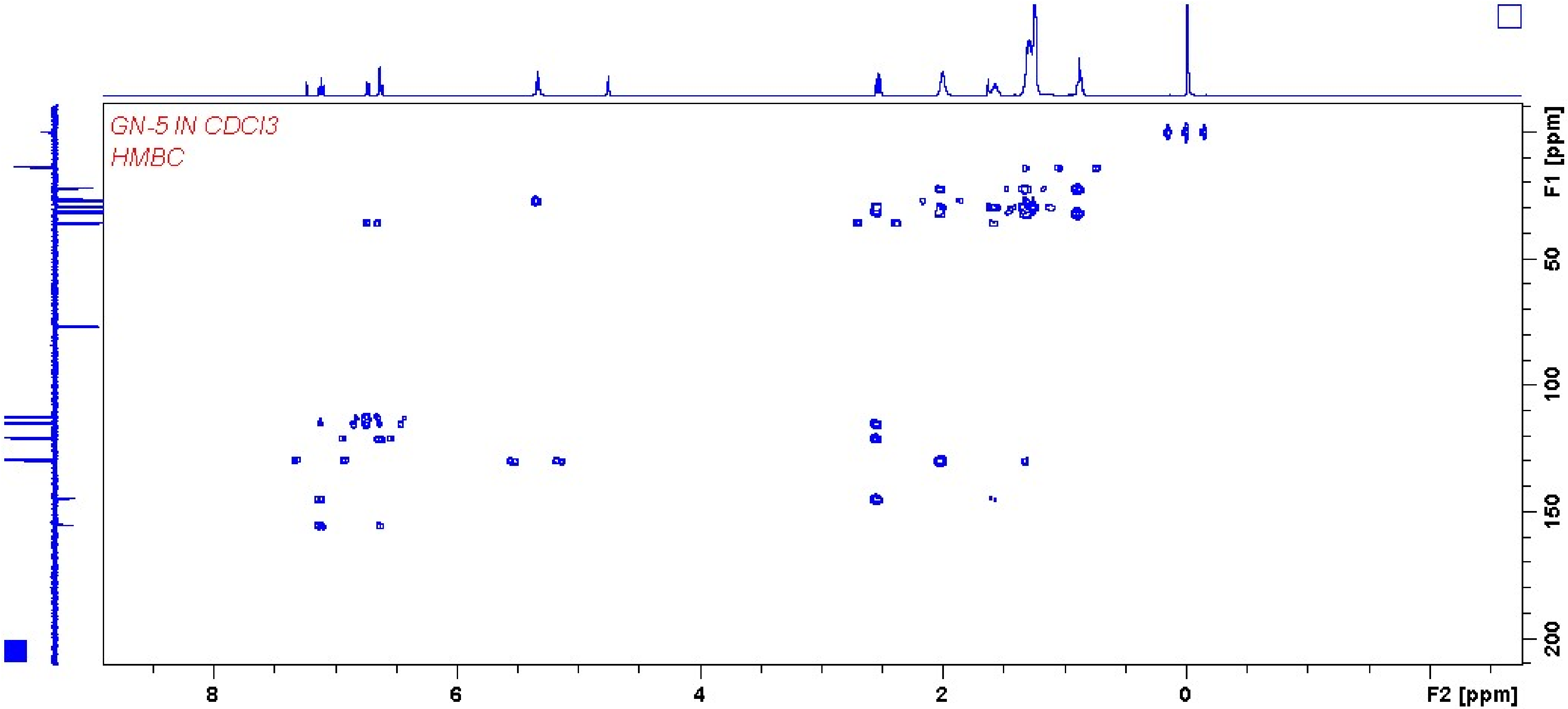

Figure S5. HMBC spectrum for 3-heptadec-12′-*Z*-enyl phenol (**1**)

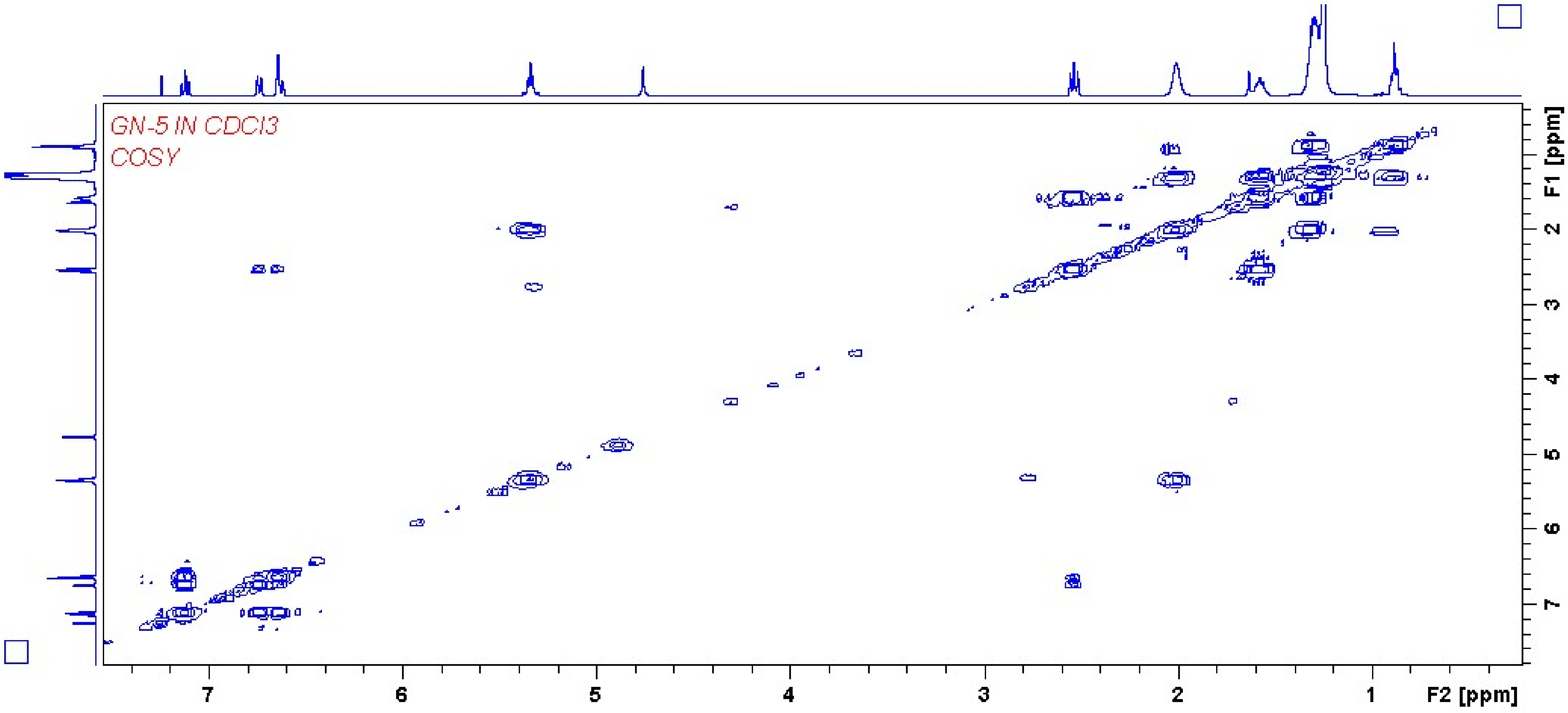

Figure S6. COSY spectrum for 3-heptadec-12′-*Z*-enyl phenol (**1**)

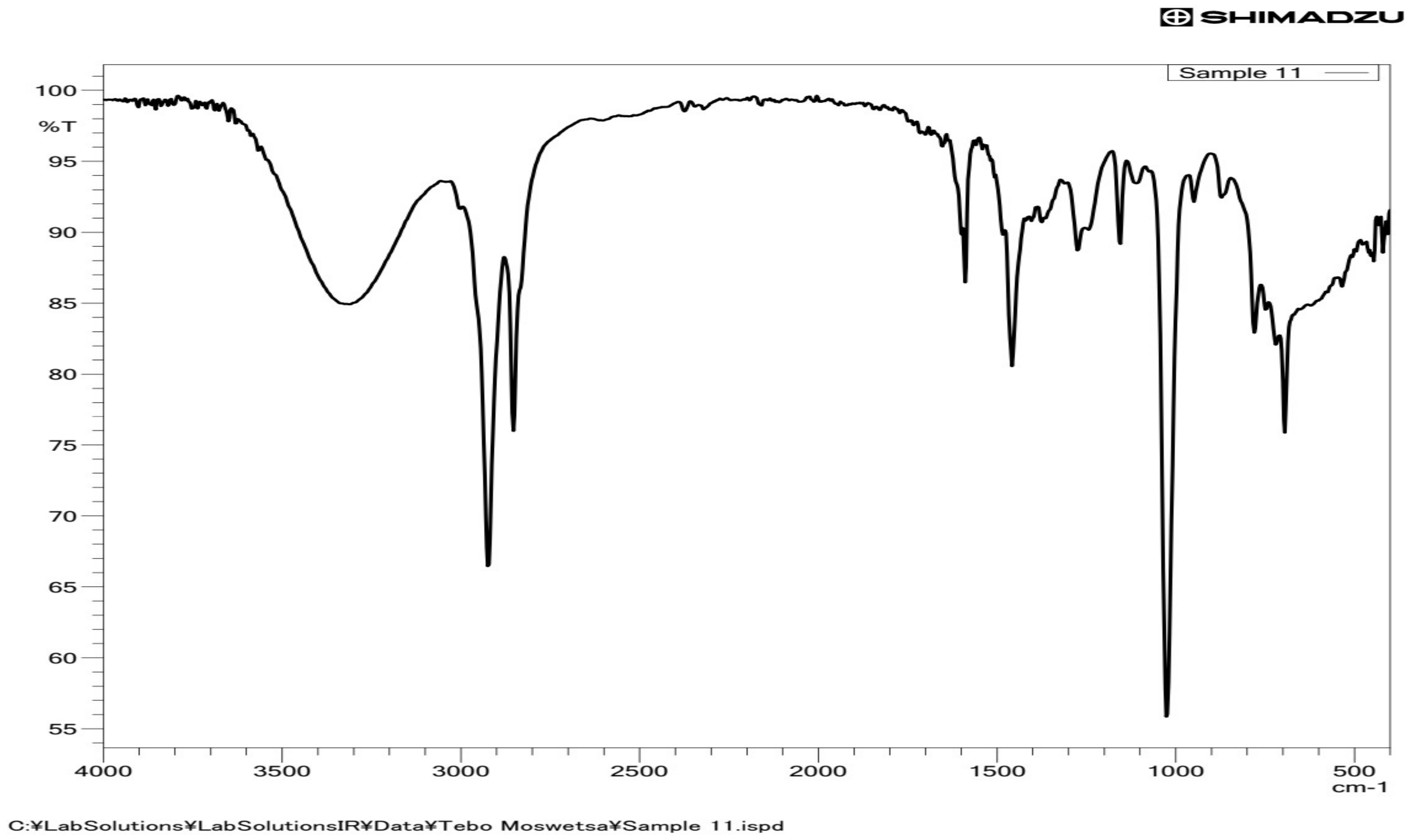

Figure S7. FTIR spectrum for 3-heptadec-12′-*Z*-enyl phenol (**1**)

Elemental Composition Report

Single Mass Analysis

Tolerance = 10.0 mDa / DBE: min = -1.5, max = 100.0
Element prediction: Off
Number of isotope peaks used for i-FIT = 3

Monoisotopic Mass, Even Electron Ions
56 formula(e) evaluated with 1 results within limits (up to 10 best isotopic matches for each mass)
Elements Used:
C: 1-50 H: 1-100 O: 1-20

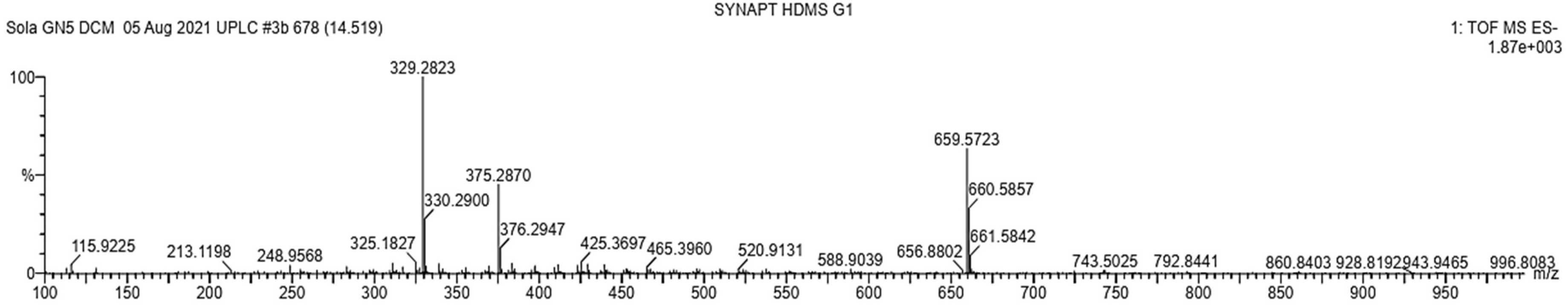

Minimum:				-1.5			
Maximum:		10.0	10.0	100.0			
Mass	Calc. Mass	mDa	PPM	DBE	i-FIT	i-FIT (Norm)	Formula
329.2823	329.2844	-2.1	-6.4	5.5	43.4	0.0	C23 H37 O

Figure S8. HR-ESI-MS for 3-heptadec-12′-*Z*-enyl phenol (**1**)

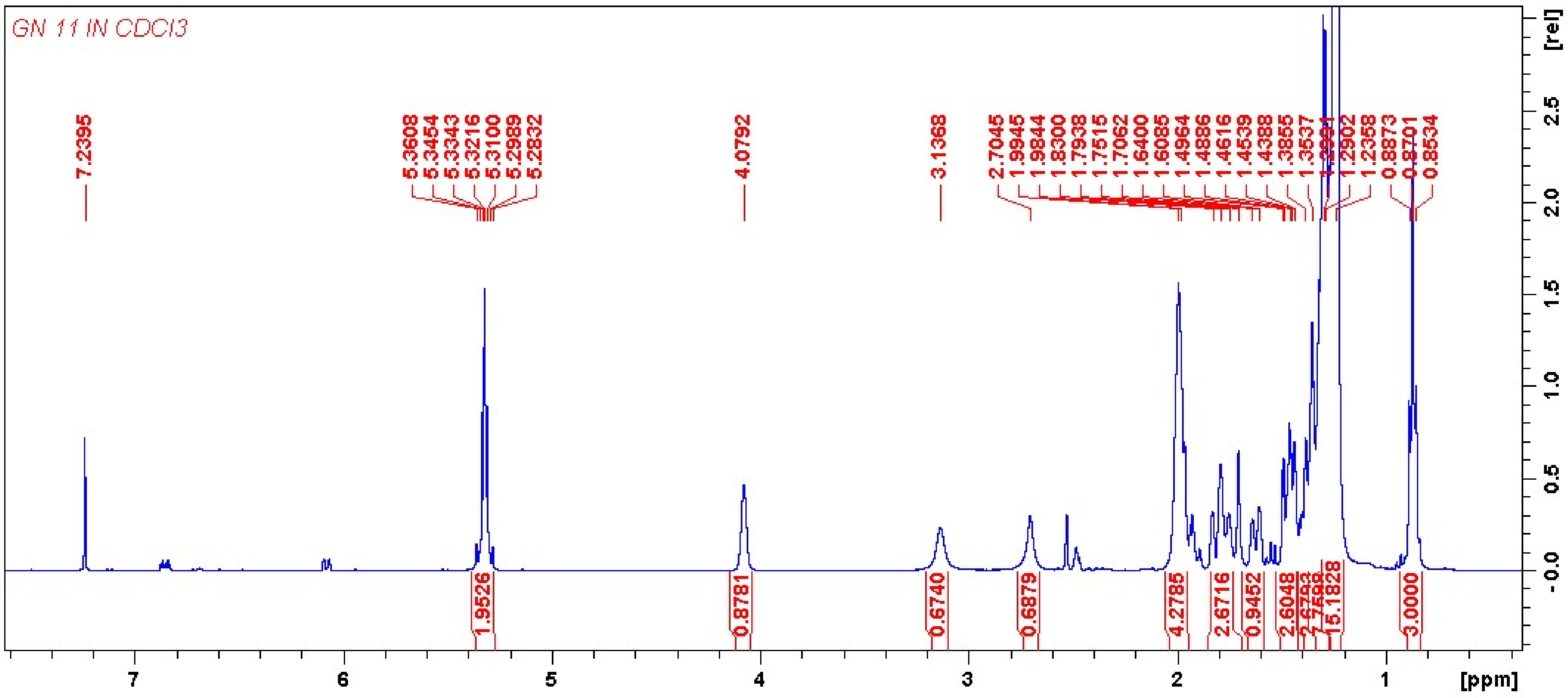

Figure S9. ¹H NMR spectrum for (1*R*,3*R*)-1,3-dihydroxy-3-[heptadec-12′(*Z*)-enyl]cyclohexane (**2**)

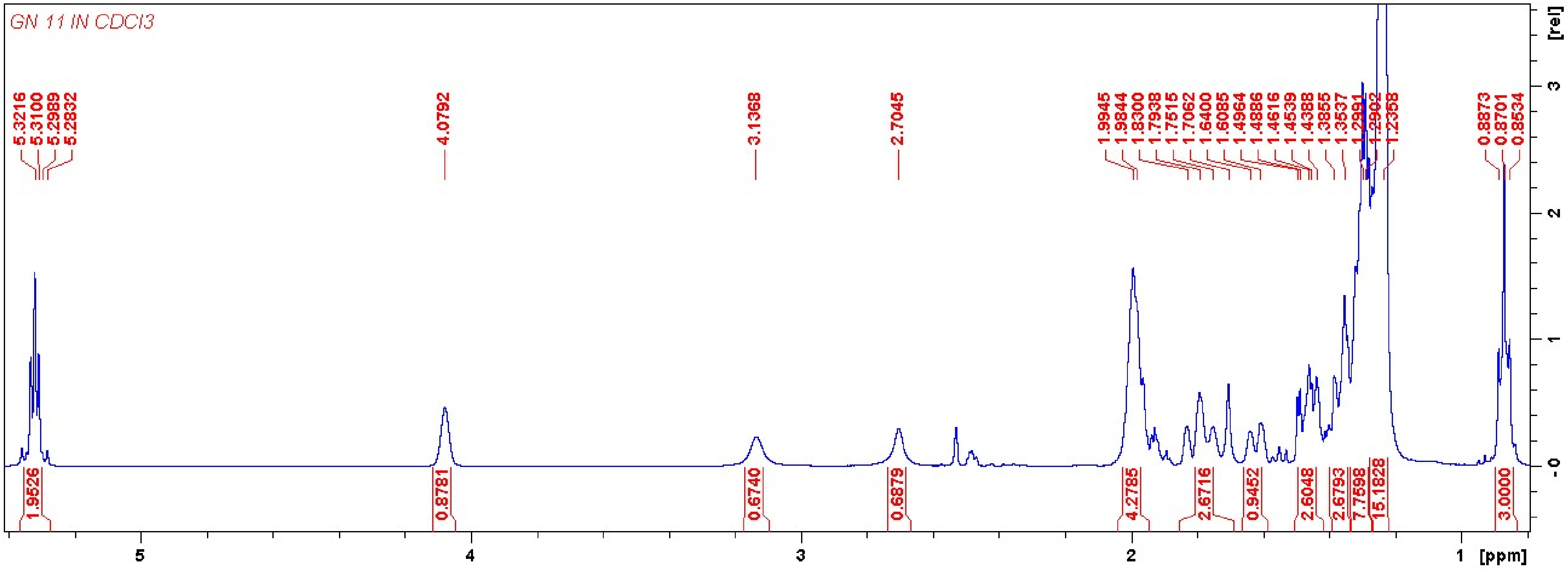

Figure S10. Expanded ^{1}H NMR spectrum for (1*R*,3*R*)-1,3-dihydroxy-3-[heptadec-12′(*Z*)-enyl]cyclohexane (**2**)

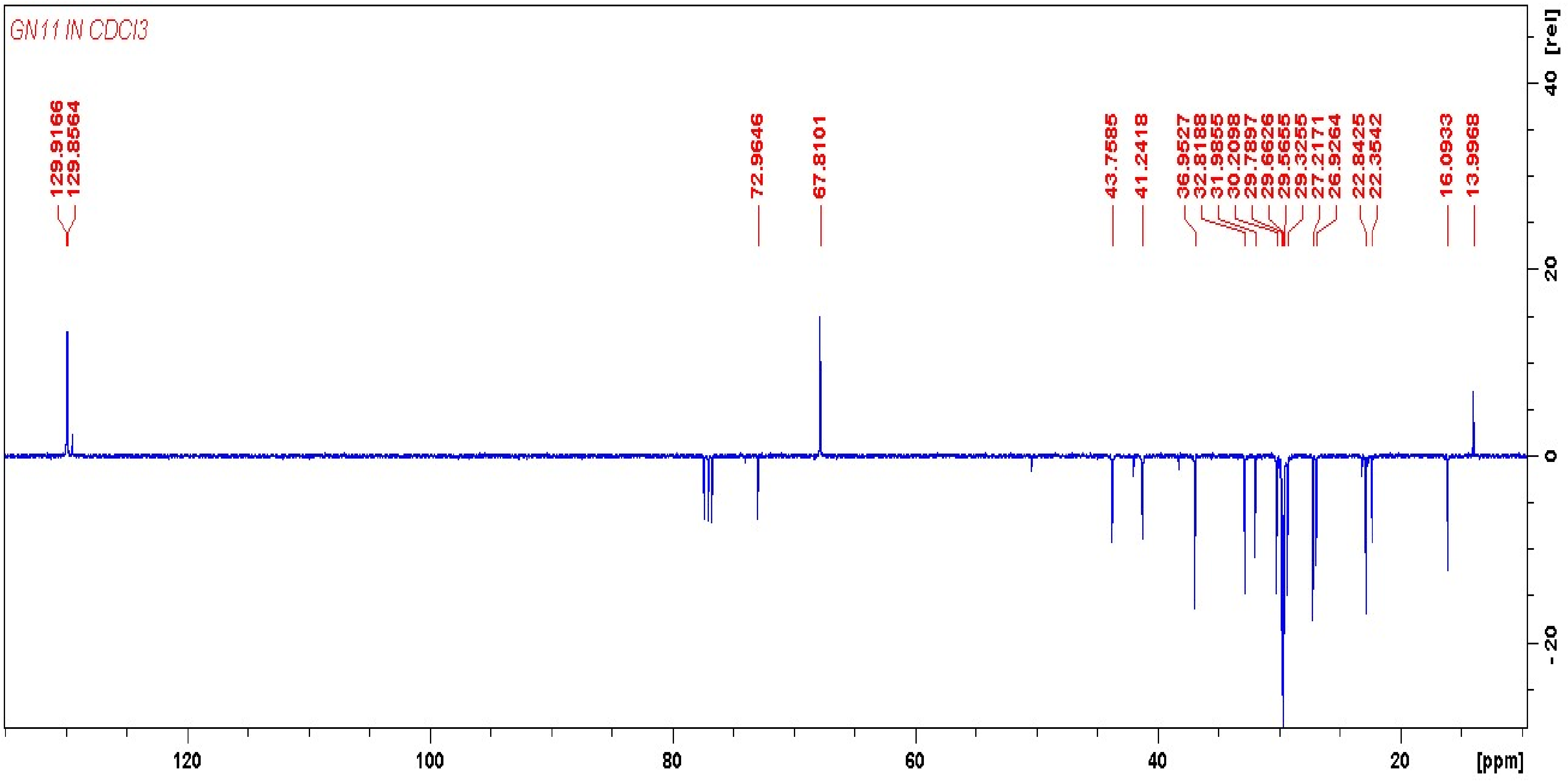

Figure S11. APT spectrum for (1*R*,3*R*)-1,3-dihydroxy-3-[heptadec-12′(*Z*)-enyl]cyclohexane (**2**)

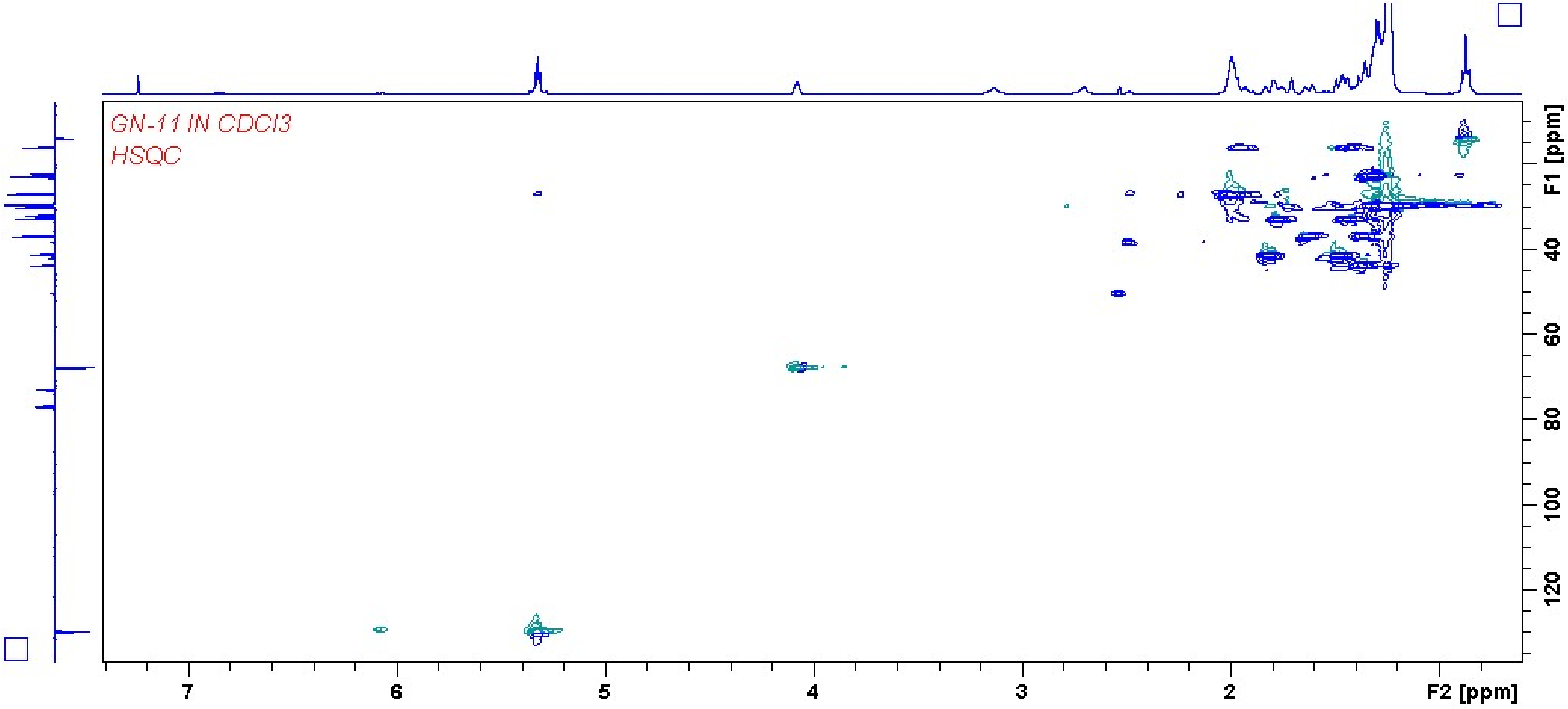

Figure S12. HSQC spectrum for (1*R*,3*R*)-1,3-dihydroxy-3-[heptadec-12′(*Z*)-enyl]cyclohexane (**2**)

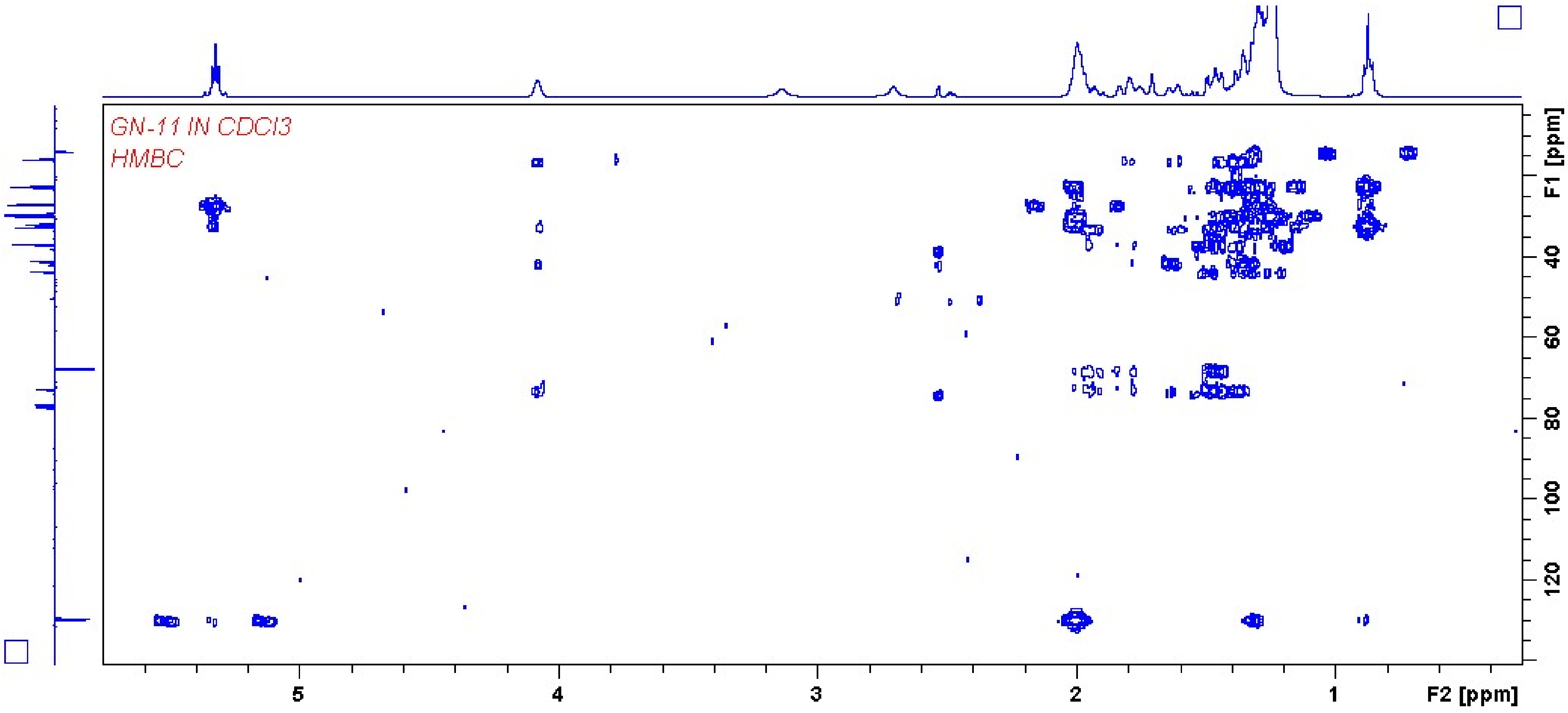

Figure S13. HMBC spectrum for (1*R*,3*R*)-1,3-dihydroxy-3-[heptadec-12′(*Z*)-enyl]cyclohexane (**2**)

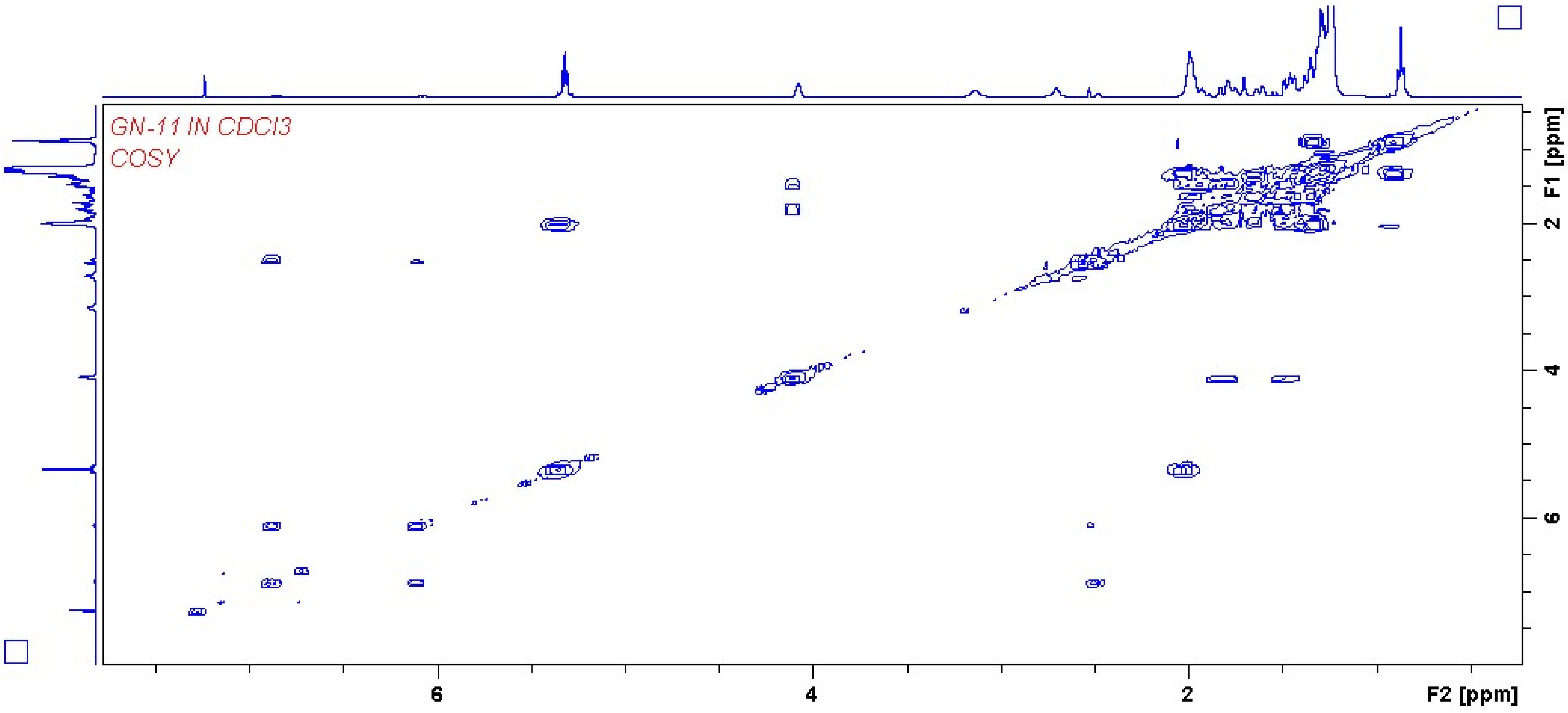

Figure S14. COSY spectrum for (1*R*,3*R*)-1,3-dihydroxy-3-[heptadec-12′(*Z*)-enyl]cyclohexane (**2**)

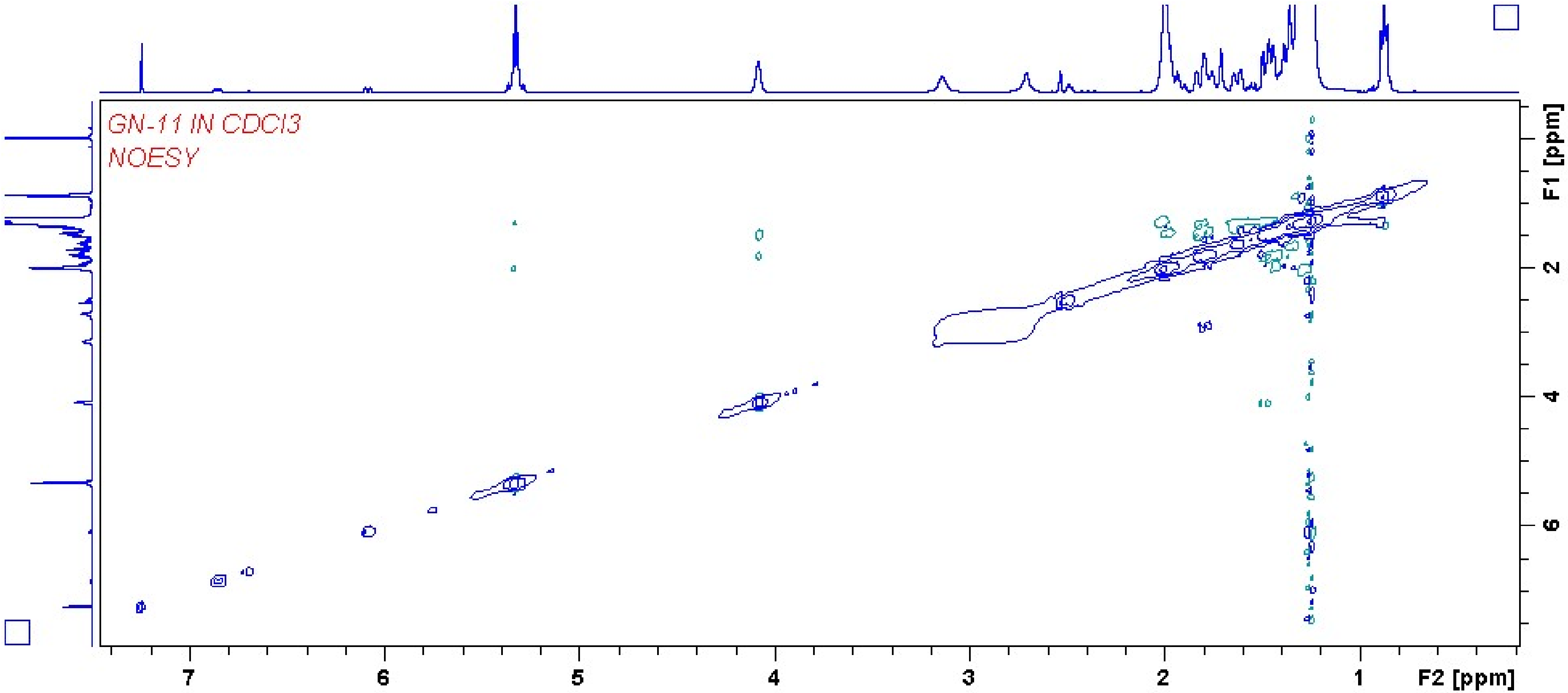

Figure S15. NOESY spectrum for (1*R*,3*R*)-1,3-dihydroxy-3-[heptadec-12′(*Z*)-enyl]cyclohexane (**2**)

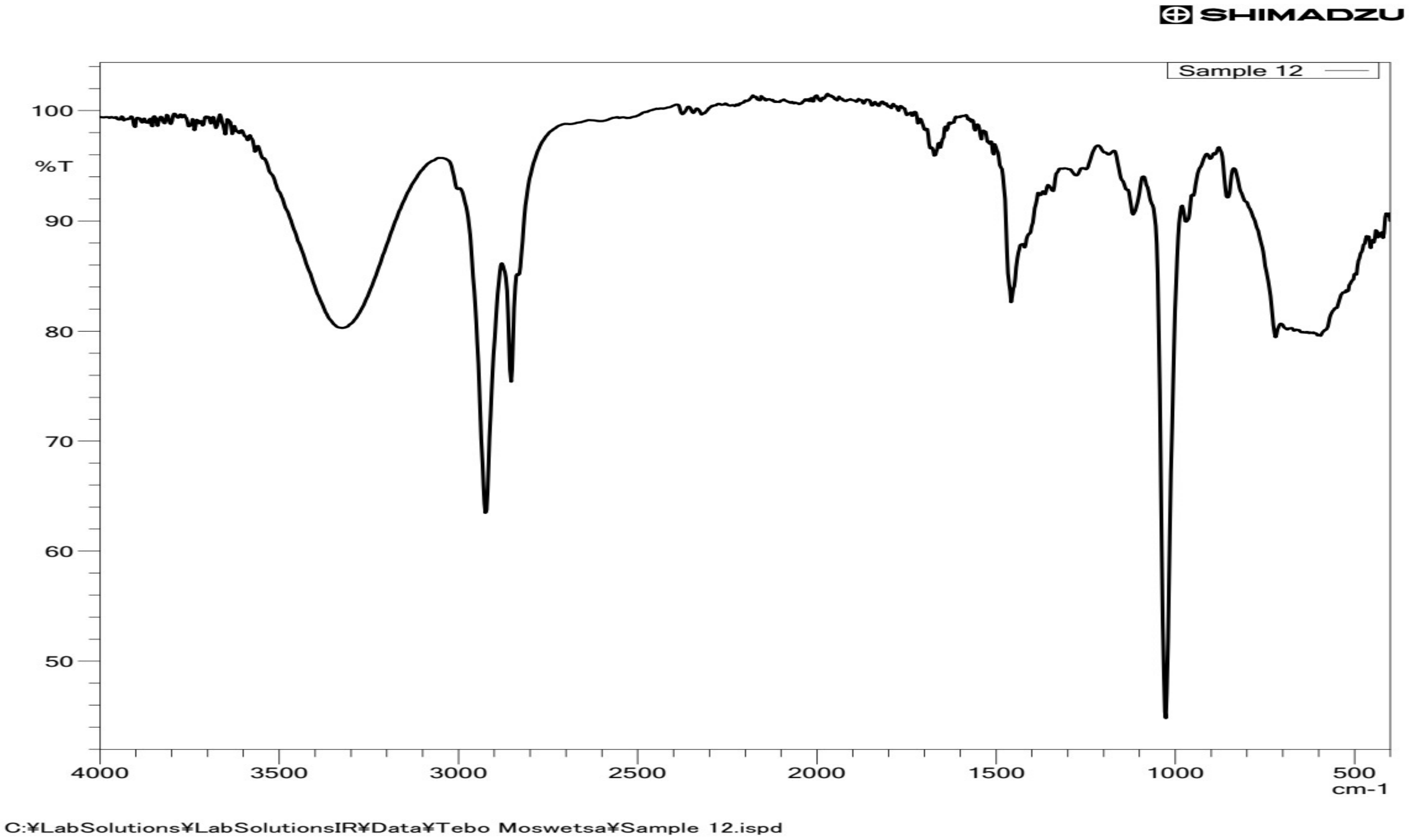

Figure S16. FTIR spectrum for (1*R*,3*R*)-1,3-dihydroxy-3-[heptadec-12′(*Z*)-enyl]cyclohexane (**2**)

Elemental Composition Report

Single Mass Analysis

Tolerance = 10.0 mDa / DBE: min = -1.5, max = 100.0
Element prediction: Off
Number of isotope peaks used for i-FIT = 3

Monoisotopic Mass, Even Electron Ions
18 formula(e) evaluated with 2 results within limits (up to 10 best isotopic matches for each mass)
Elements Used:
C: 1-40 H: 1-100 O: 1-2 Na: 0-1

SYNAPT HDMS G1

Sola GN11 DCM 05 Aug 2021 UPLC #3a 666 (13.801)

1: TOF MS ES+
2.05e+004

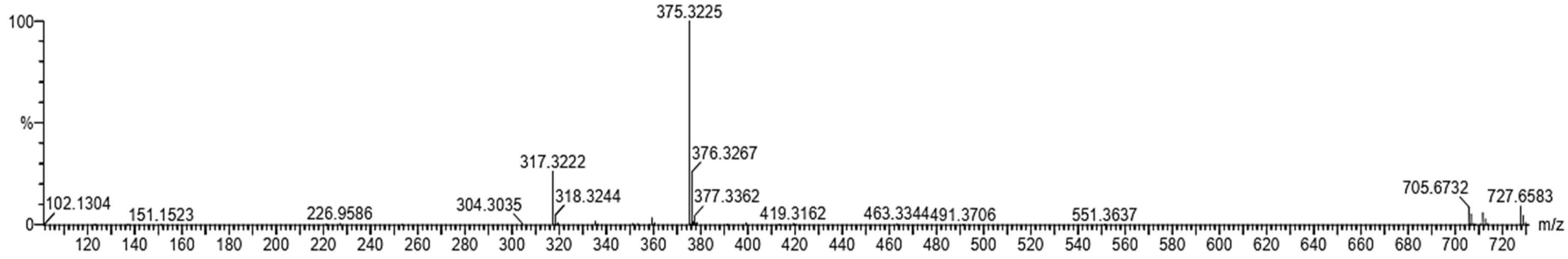

Minimum:				-1.5			
Maximum:		10.0	10.0	100.0			
Mass	Calc. Mass	mDa	PPM	DBE	i-FIT	i-FIT (Norm)	Formula
375.3225	375.3239	-1.4	-3.7	1.5	102.8	0.0	C23 H44 O2 Na

Figure S17. HR-ESI-MS for (1*R*,3*R*)-1,3-dihydroxy-3-[heptadec-12′(*Z*)-enyl]cyclohexane (**2**)

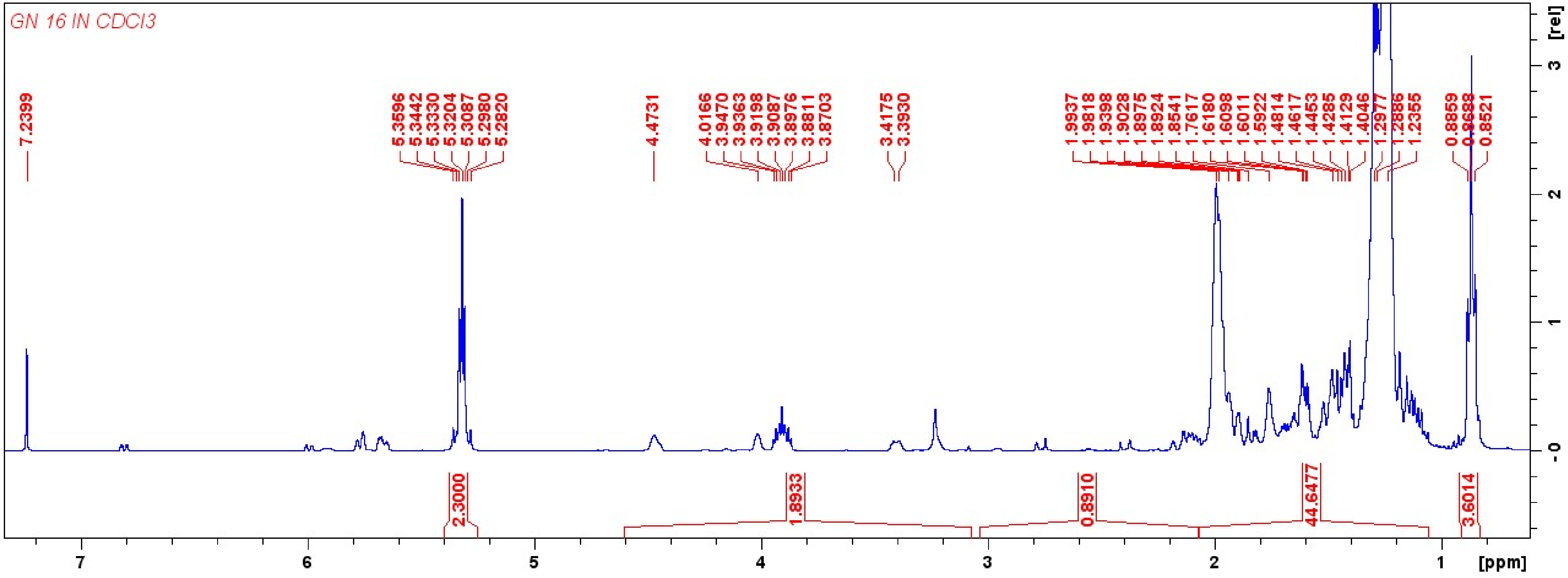

Figure S18. ^{1}H NMR spectrum for (1*S*,2*S*,3*S*,4*S*,5*R*)-1,2,3,4,5-pentahydroxy-5-[octadec-13′(*Z*)-enyl]cyclohexane (**3**)

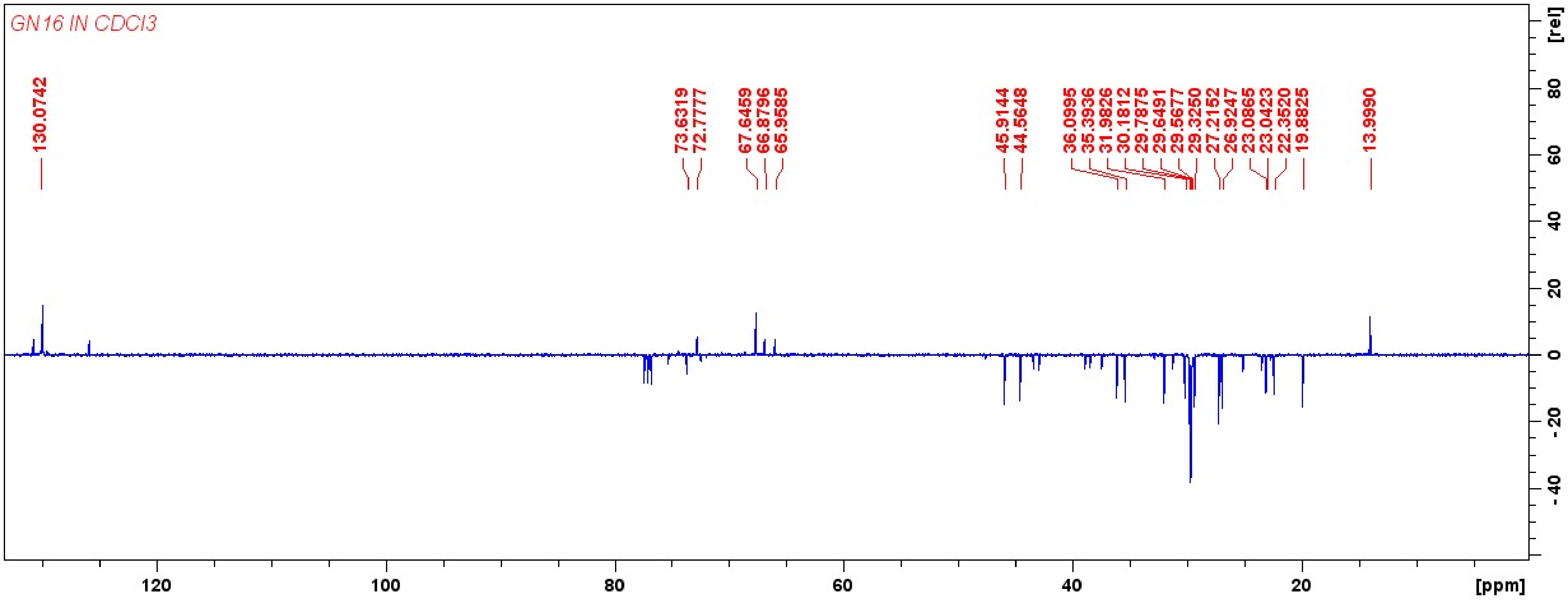

Figure S19. APT spectrum for (1*S*,2*S*,3*S*,4*S*,5*R*)-1,2,3,4,5-pentahydroxy-5-[octadec-13′(*Z*)-enyl]cyclohexane (**3**)

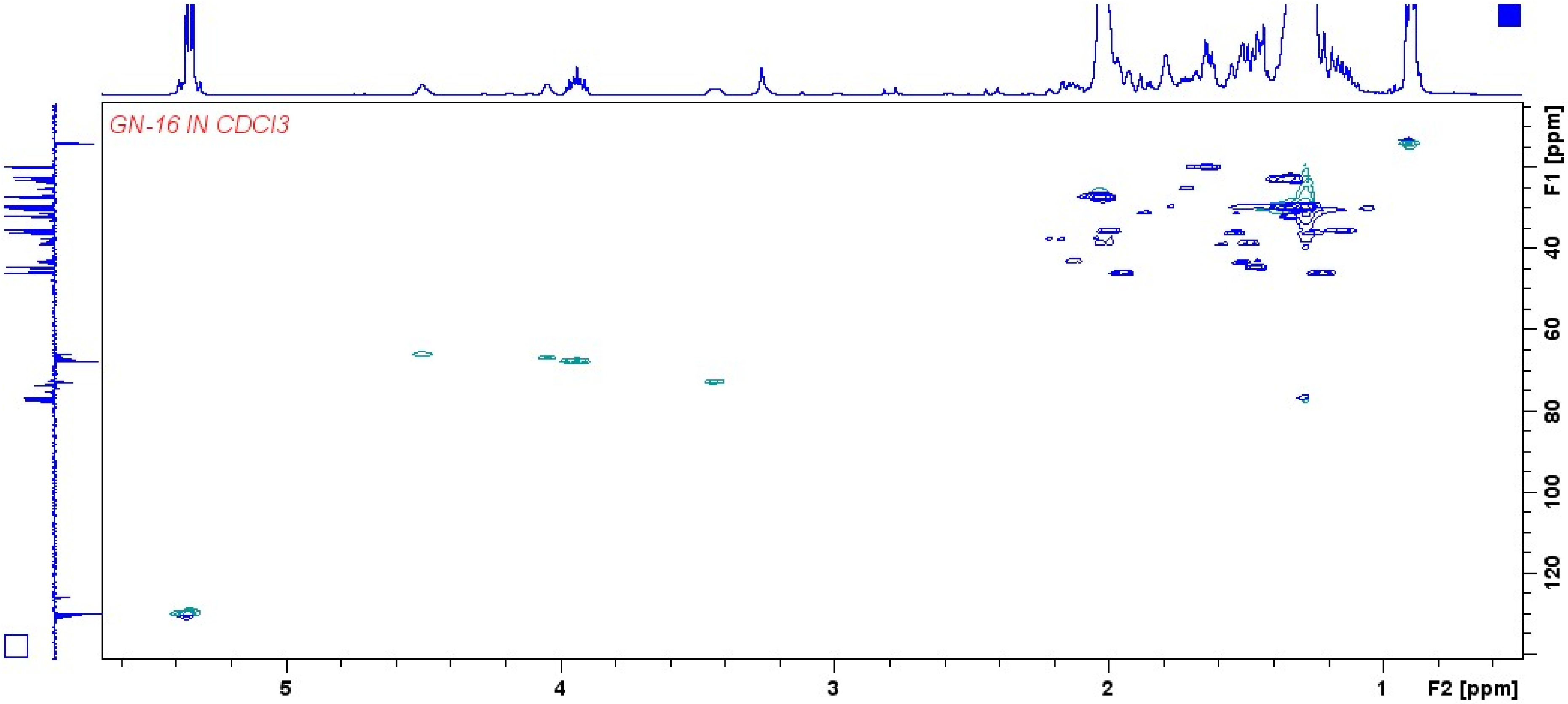

Figure S20. HSQC spectrum for (1*S*,2*S*,3*S*,4*S*,5*R*)-1,2,3,4,5-pentahydroxy-5-[octadec-13′(*Z*)-enyl]cyclohexane (**3**)

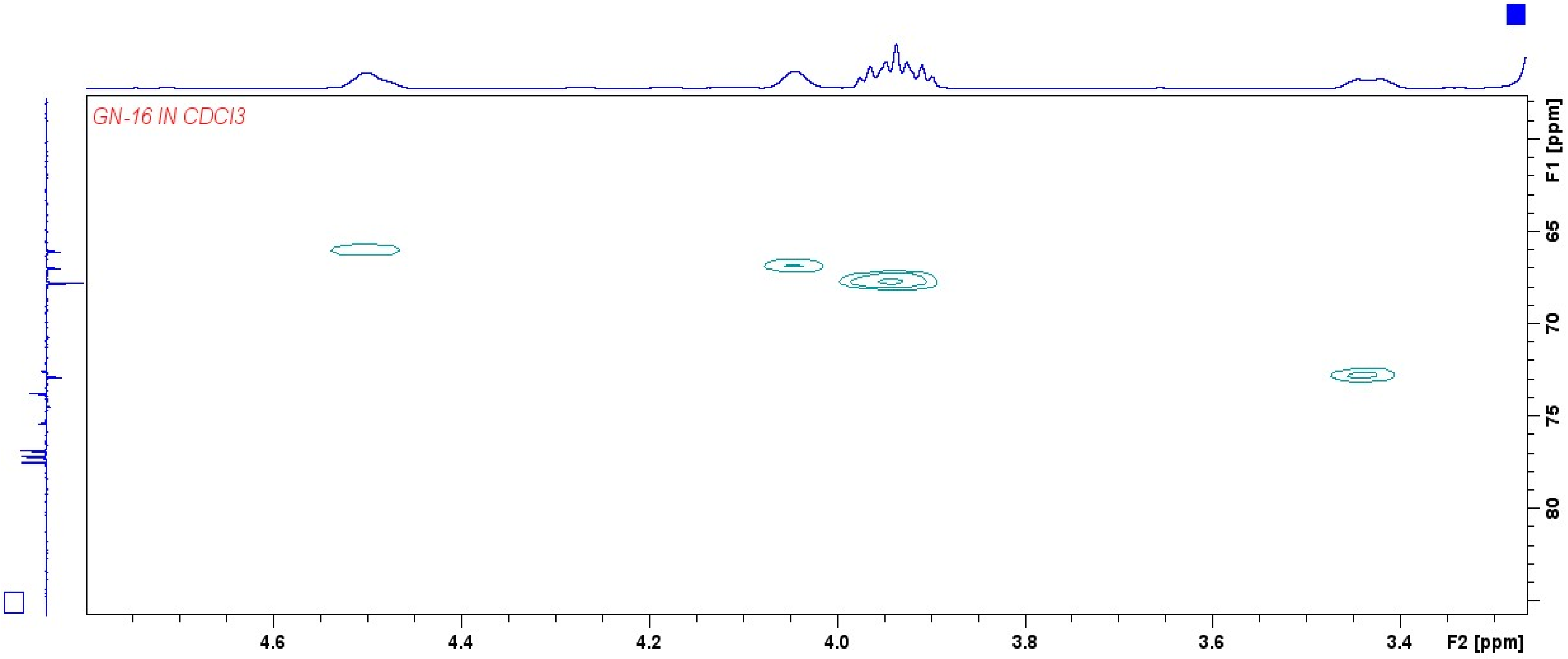

Figure S21. Expanded HSQC for (1*S*,2*S*,3*S*,4*S*,5*R*)-1,2,3,4,5-pentahydroxy-5-[octadec-13′(*Z*)-enyl]cyclohexane (**3**)

(The weak ^{1}H *NMR resonances of the oxygenated methine protons necessitated the expansion to clarify their correlations with their respective carbons*)

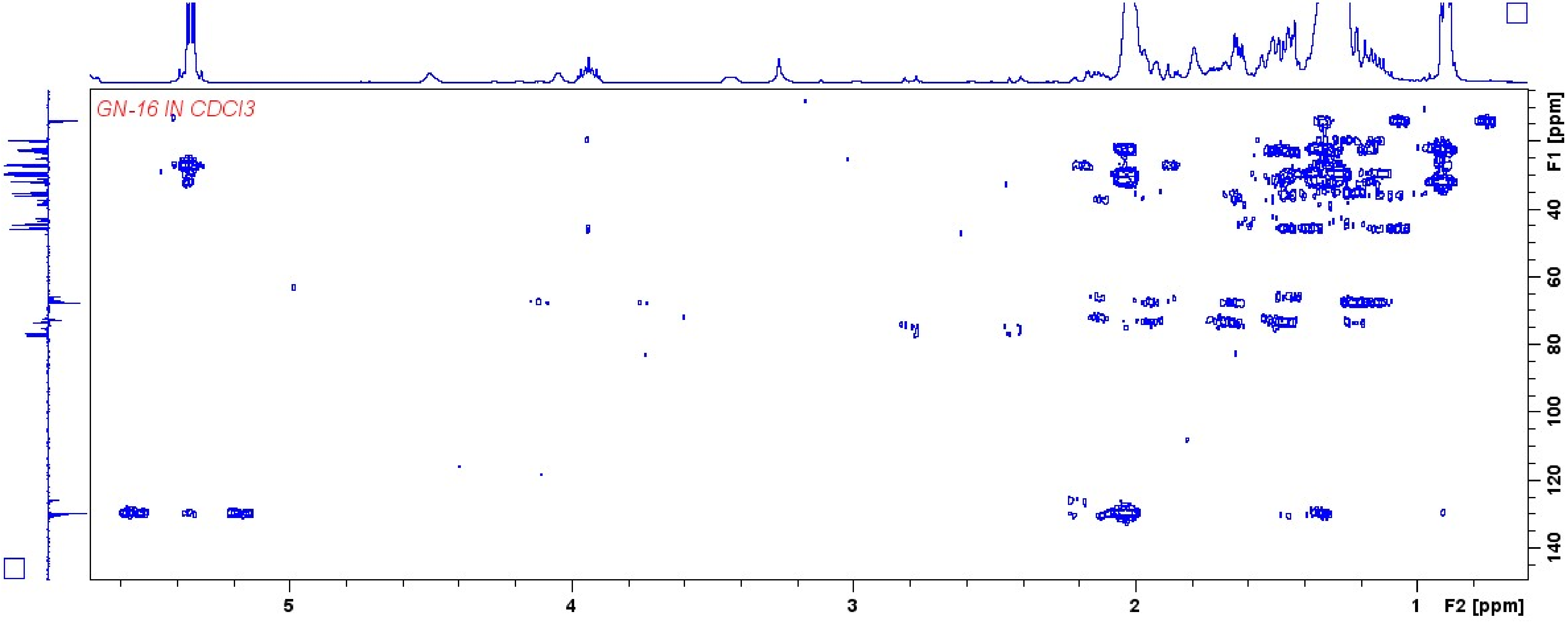

Figure S22. HMBC spectrum for (1*S*,2*S*,3*S*,4*S*,5*R*)-1,2,3,4,5-pentahydroxy-5-[octadec-13′(*Z*)-enyl]cyclohexane (**3**)

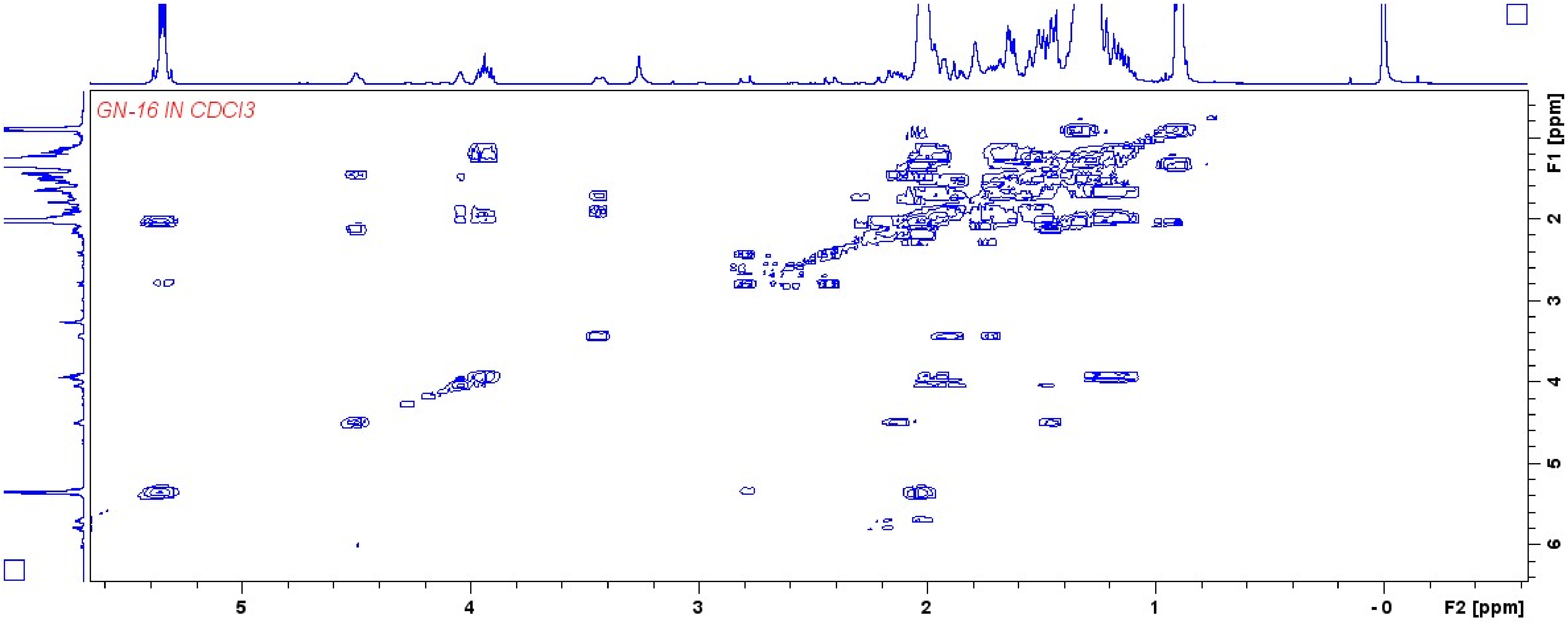

Figure S23. COSY spectrum for (1*S*,2*S*,3*S*,4*S*,5*R*)-1,2,3,4,5-pentahydroxy-5-[octadec-13′(*Z*)-enyl]cyclohexane (**3**)

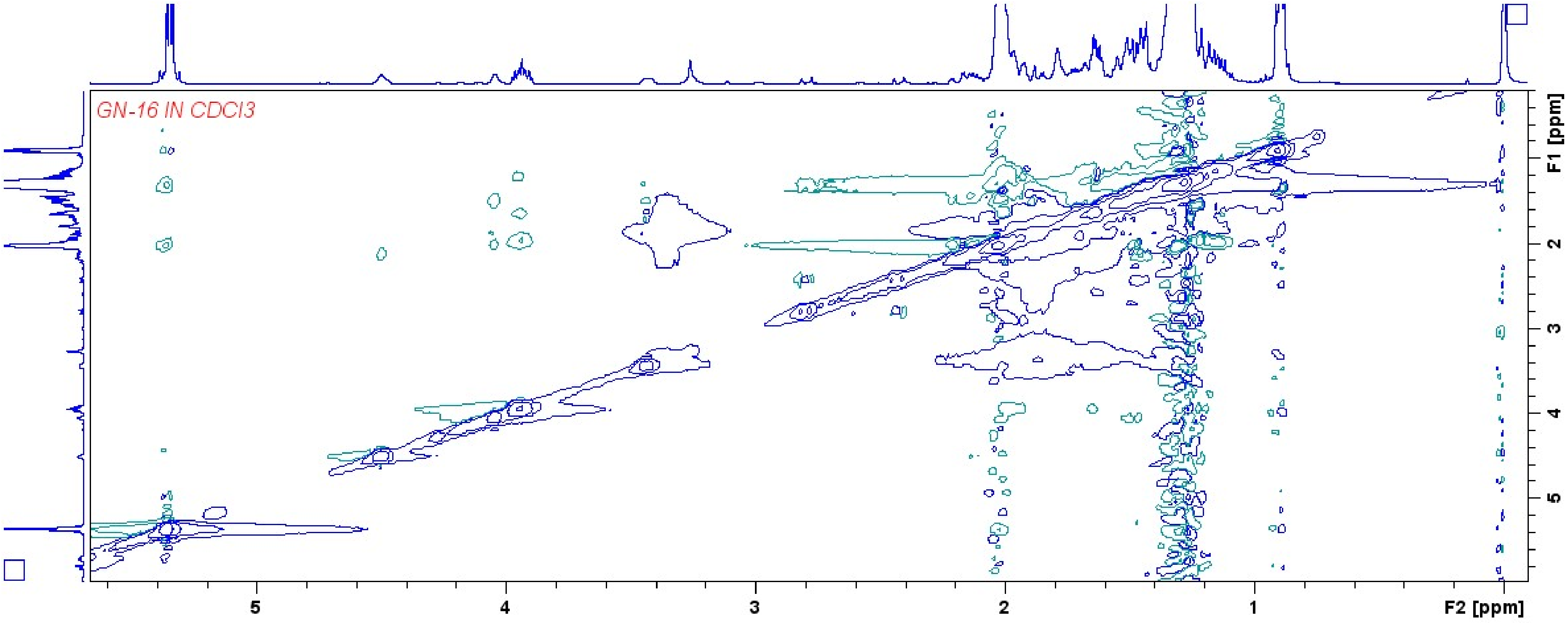

Figure S24. NOESY spectrum for (1*S*,2*S*,3*S*,4*S*,5*R*)-1,2,3,4,5-pentahydroxy-5-[octadec-13′(*Z*)-enyl]cyclohexane (**3**)

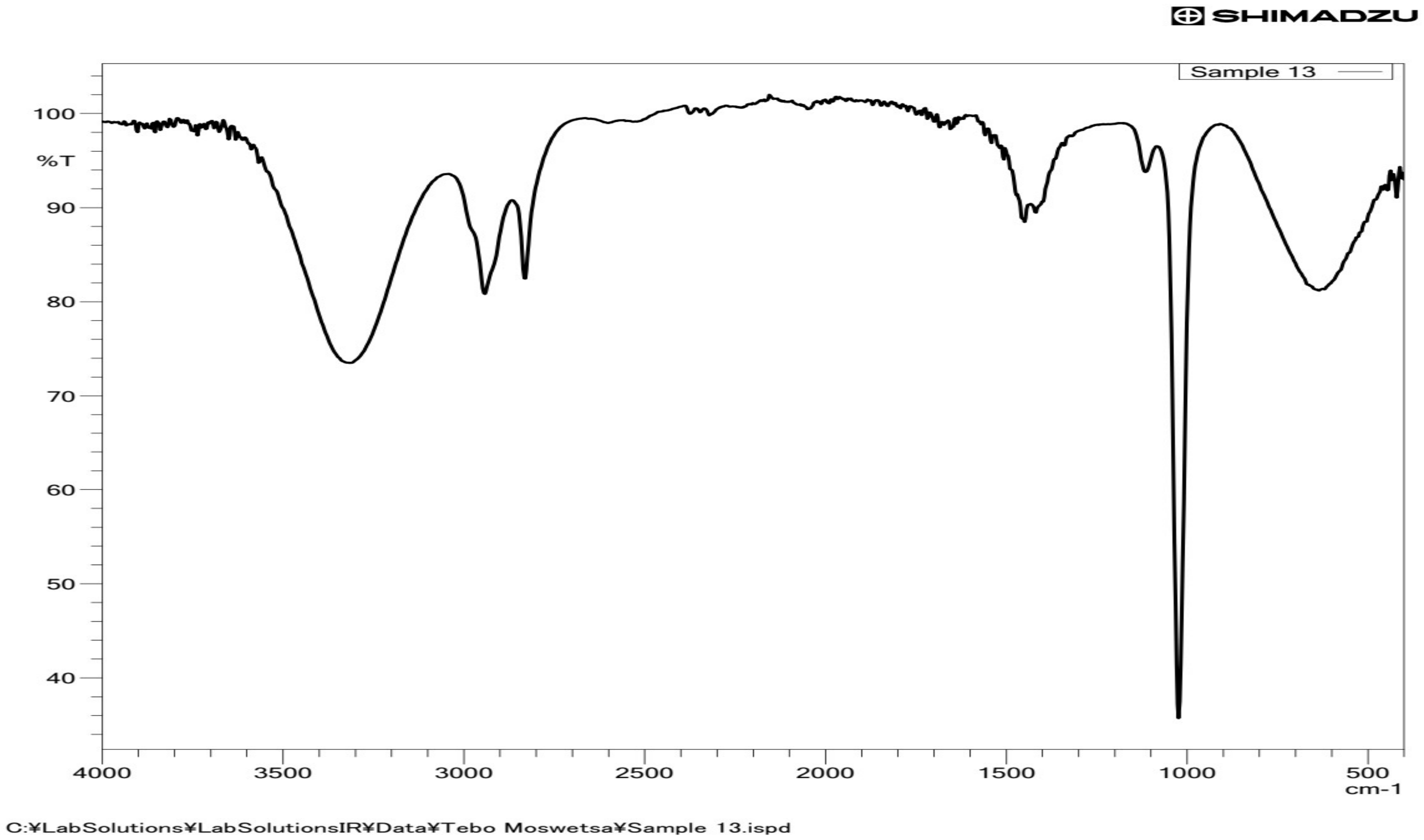

Figure S25. FTIR spectrum for (1*S*,2*S*,3*S*,4*S*,5*R*)-1,2,3,4,5-pentahydroxy-5-[octadec-13′(*Z*)-enyl]cyclohexane (**3**)

Elemental Composition Report

Single Mass Analysis

Tolerance = 10.0 mDa / DBE: min = -1.5, max = 100.0
Element prediction: Off
Number of isotope peaks used for i-FIT = 3

Monoisotopic Mass, Even Electron Ions
26 formula(e) evaluated with 1 results within limits (up to 10 best isotopic matches for each mass)
Elements Used:
C: 1-40 H: 1-100 O: 1-5

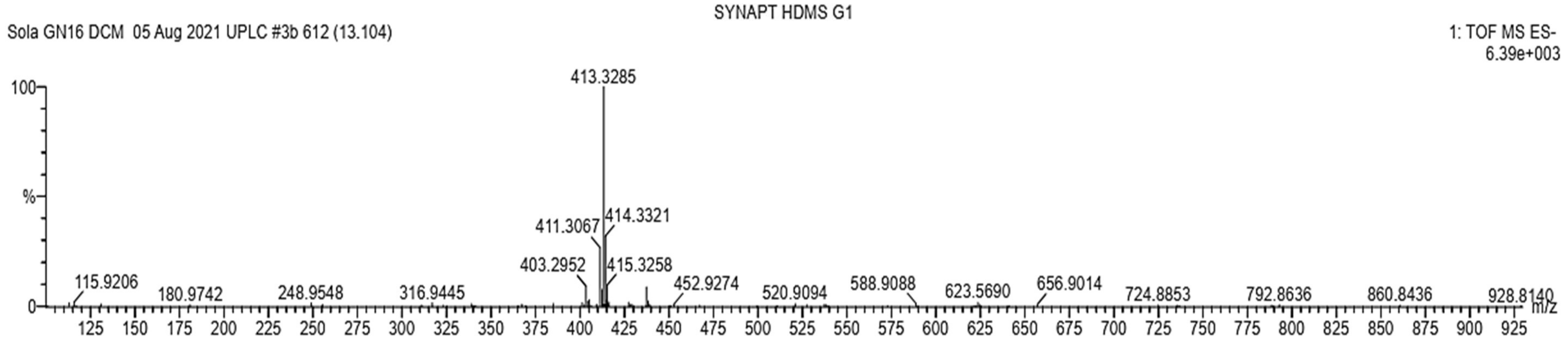

Minimum:				-1.5			
Maximum:		10.0	10.0	100.0			

Mass	Calc. Mass	mDa	PPM	DBE	i-FIT	i-FIT (Norm)	Formula
413.3285	413.3267	1.8	4.4	2.5	84.8	0.0	C24 H45 O5

Figure S26. HR-ESI-MS for (1*S*,2*S*,3*S*,4*S*,5*R*)-1,2,3,4,5-pentahydroxy-5-[octadec-13′(*Z*)-enyl]cyclohexane (**3**)

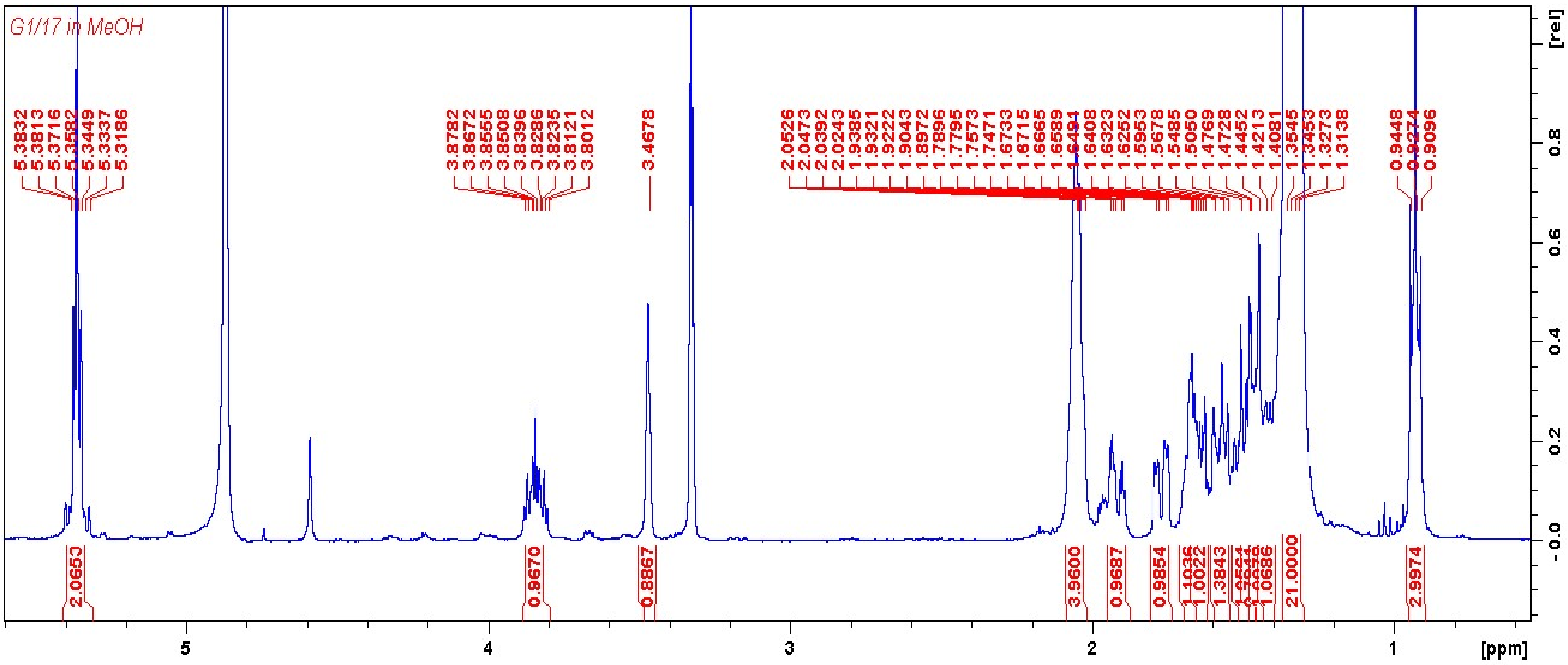

Figure S27. ^{1}H-NMR spectrum for (1*R*,2*S*,4*R*)-1,2,4-trihydroxy-4-[heptadec-12′(*Z*)-enyl]cyclohexane (**4**)

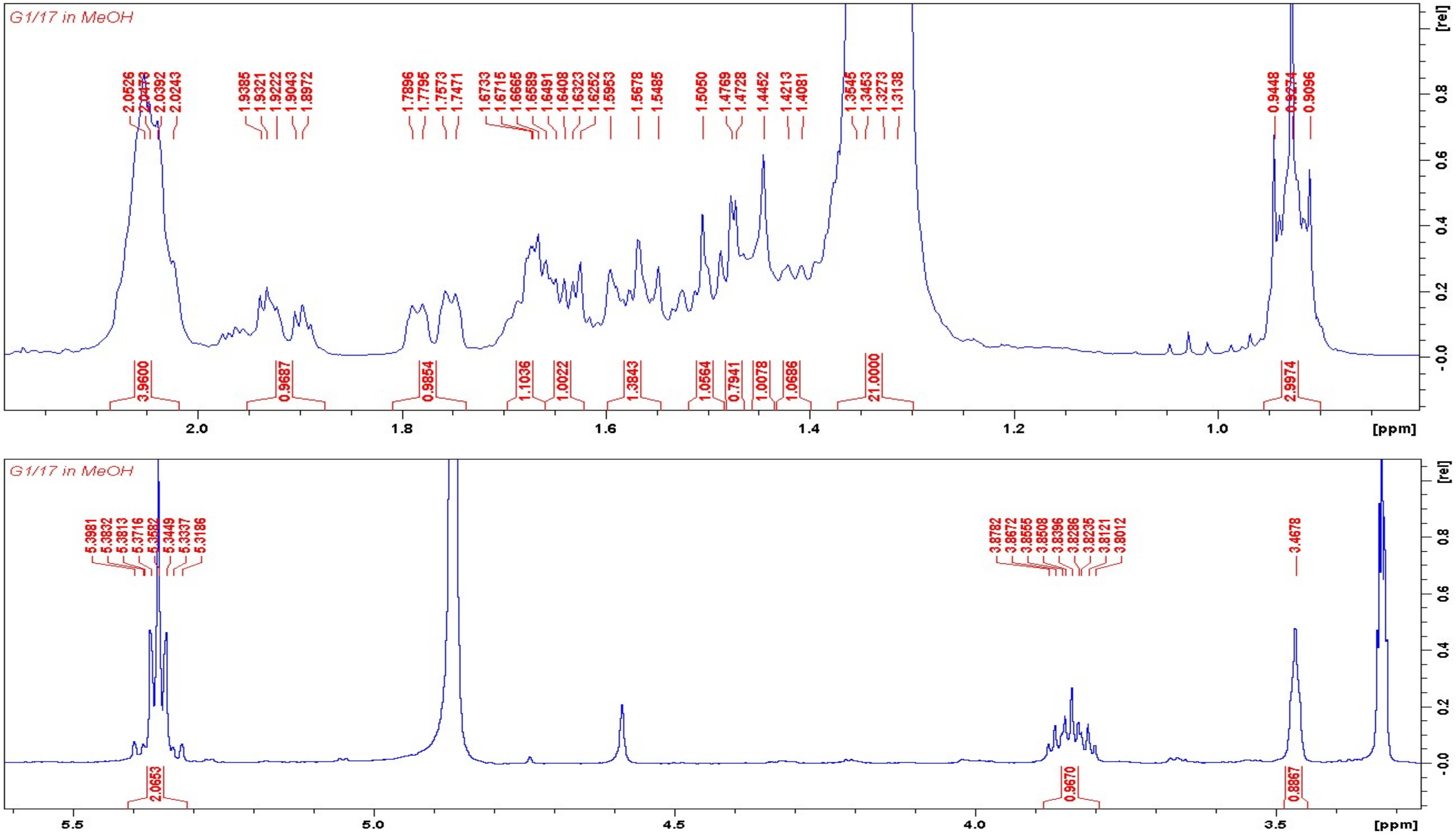

Figure S28. Expanded ^{1}H-NMR spectra for (1*R*,2*S*,4*R*)-1,2,4-trihydroxy-4-[heptadec-12′(*Z*)-enyl]cyclohexane (**4**)

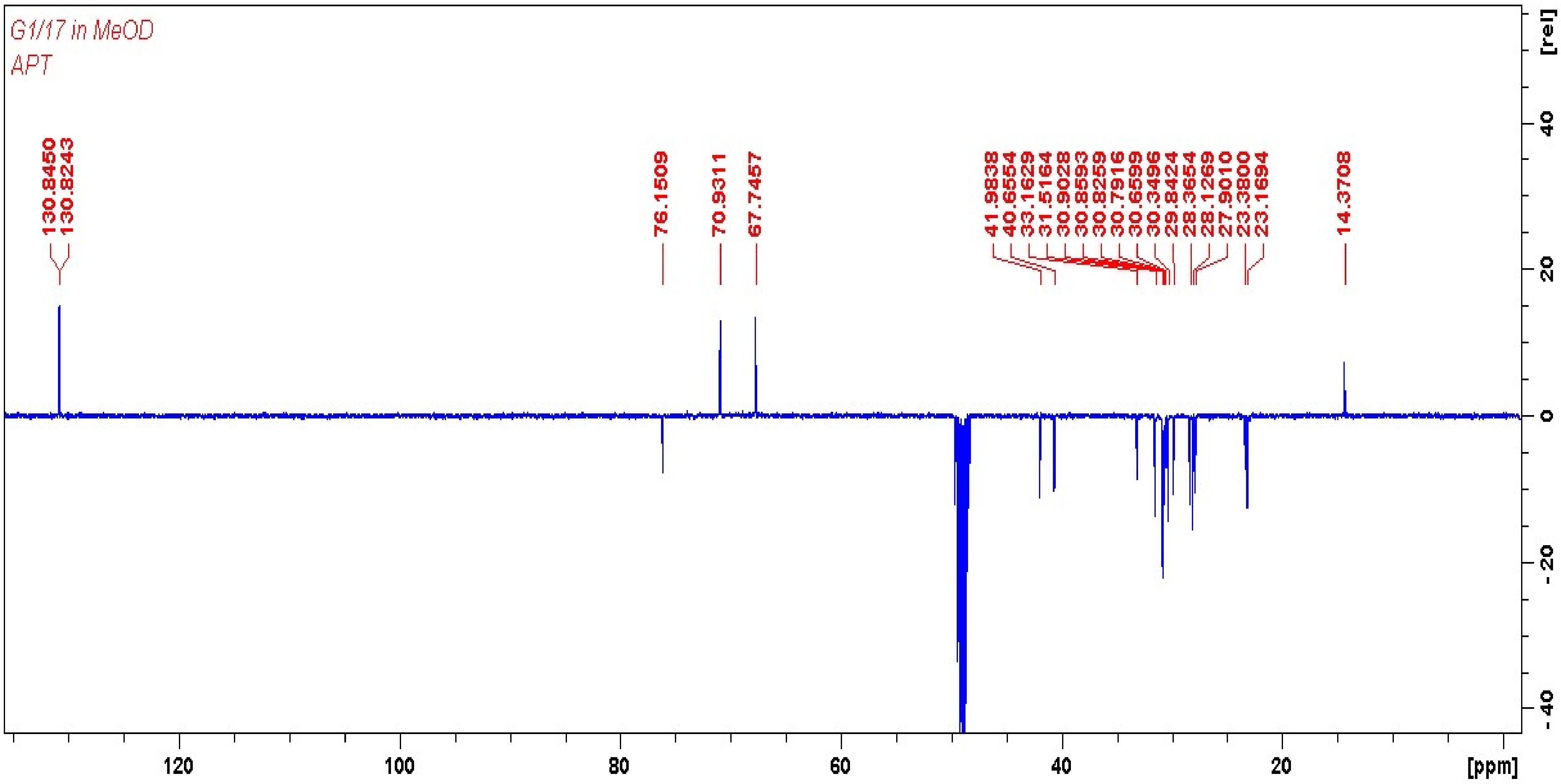

Figure S29. APT spectrum for (1*R*,2*S*,4*R*)-1,2,4-trihydroxy-4-[heptadec-12′(*Z*)-enyl]cyclohexane (**4**)

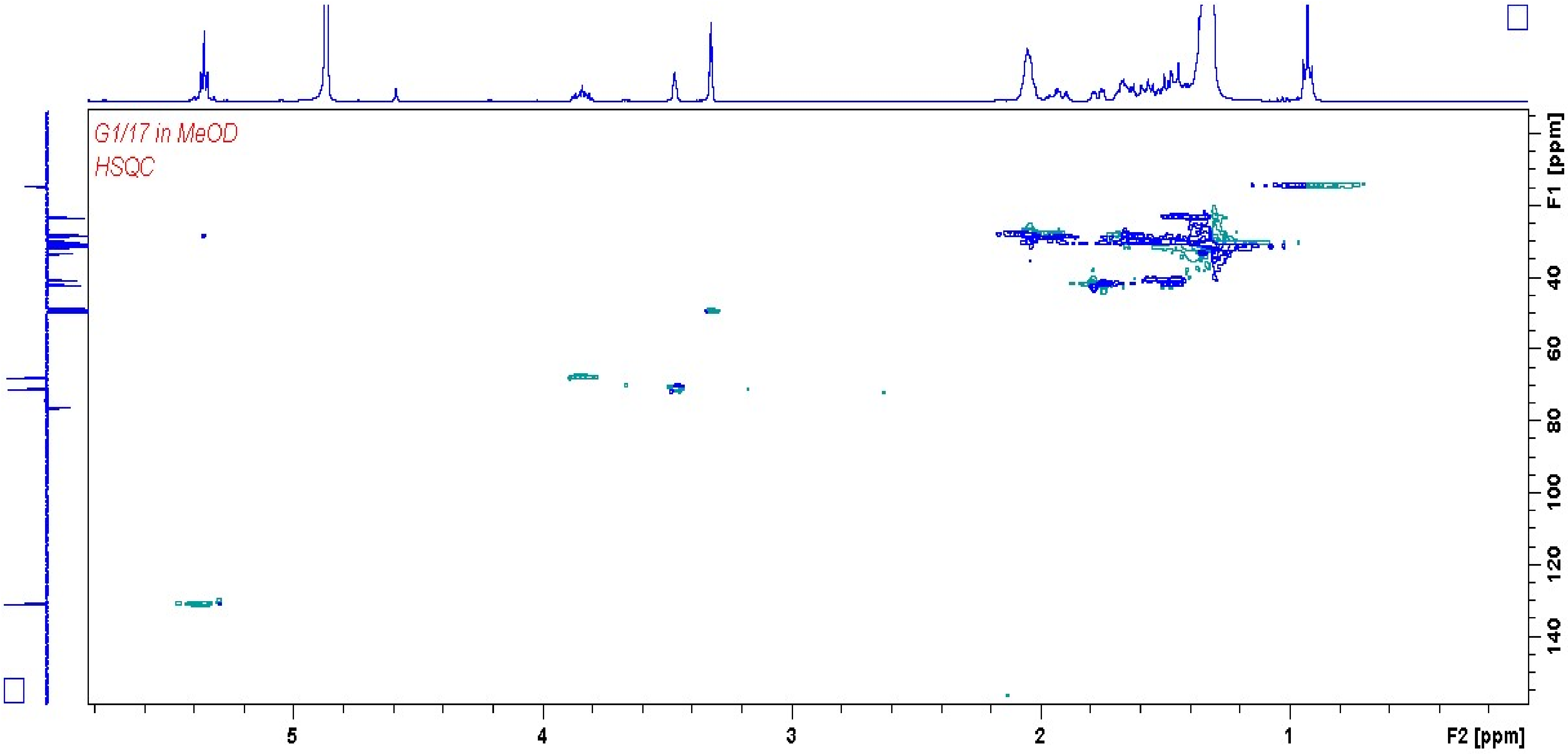

Figure S30. HSQC spectrum for (1*R*,2*S*,4*R*)-1,2,4-trihydroxy-4-[heptadec-12′(*Z*)-enyl]cyclohexane (**4**)

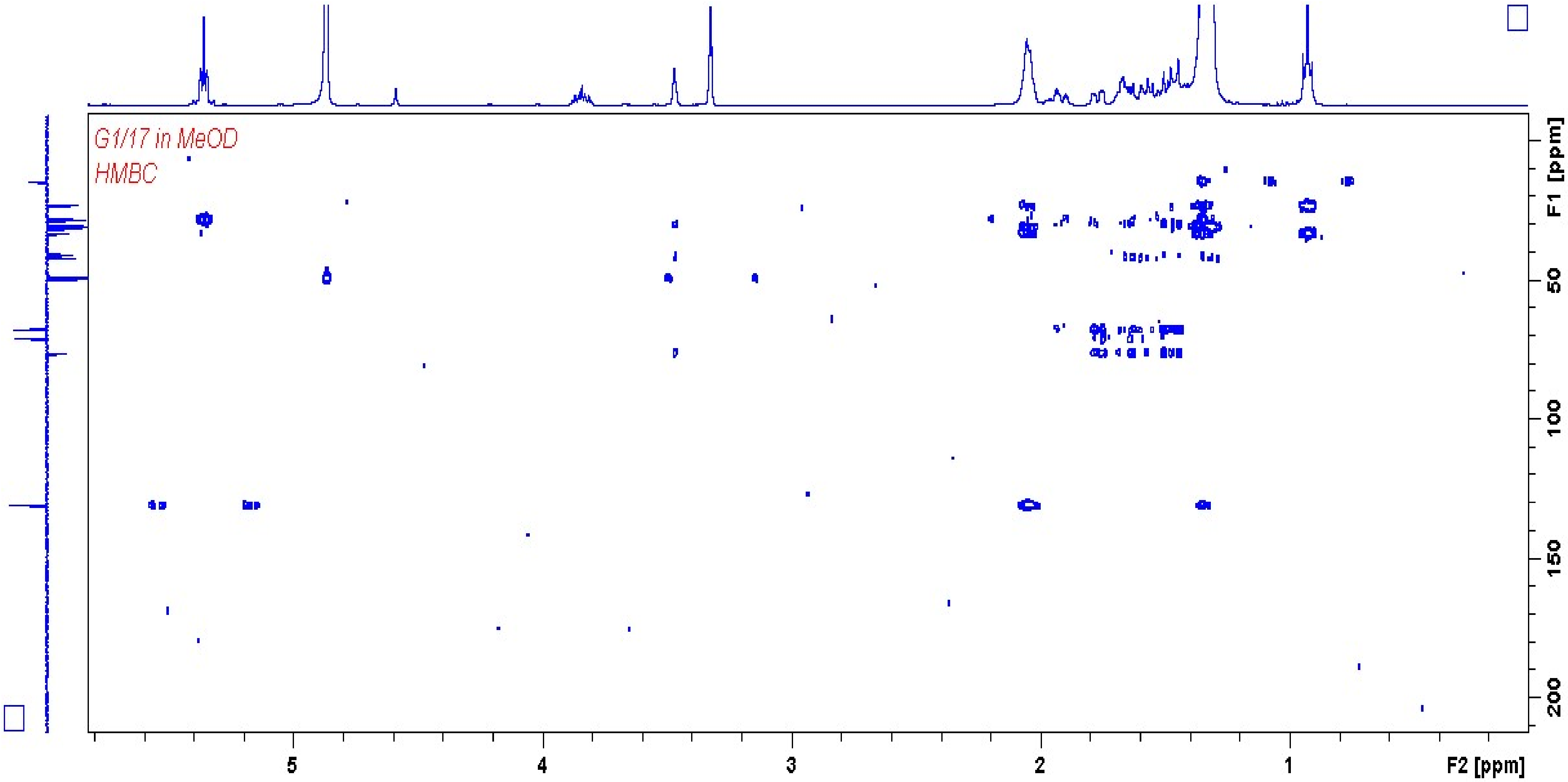

Figure S31. HMBC spectrum for (1*R*,2*S*,4*R*)-1,2,4-trihydroxy-4-[heptadec-12′(*Z*)-enyl]cyclohexane (**4**)

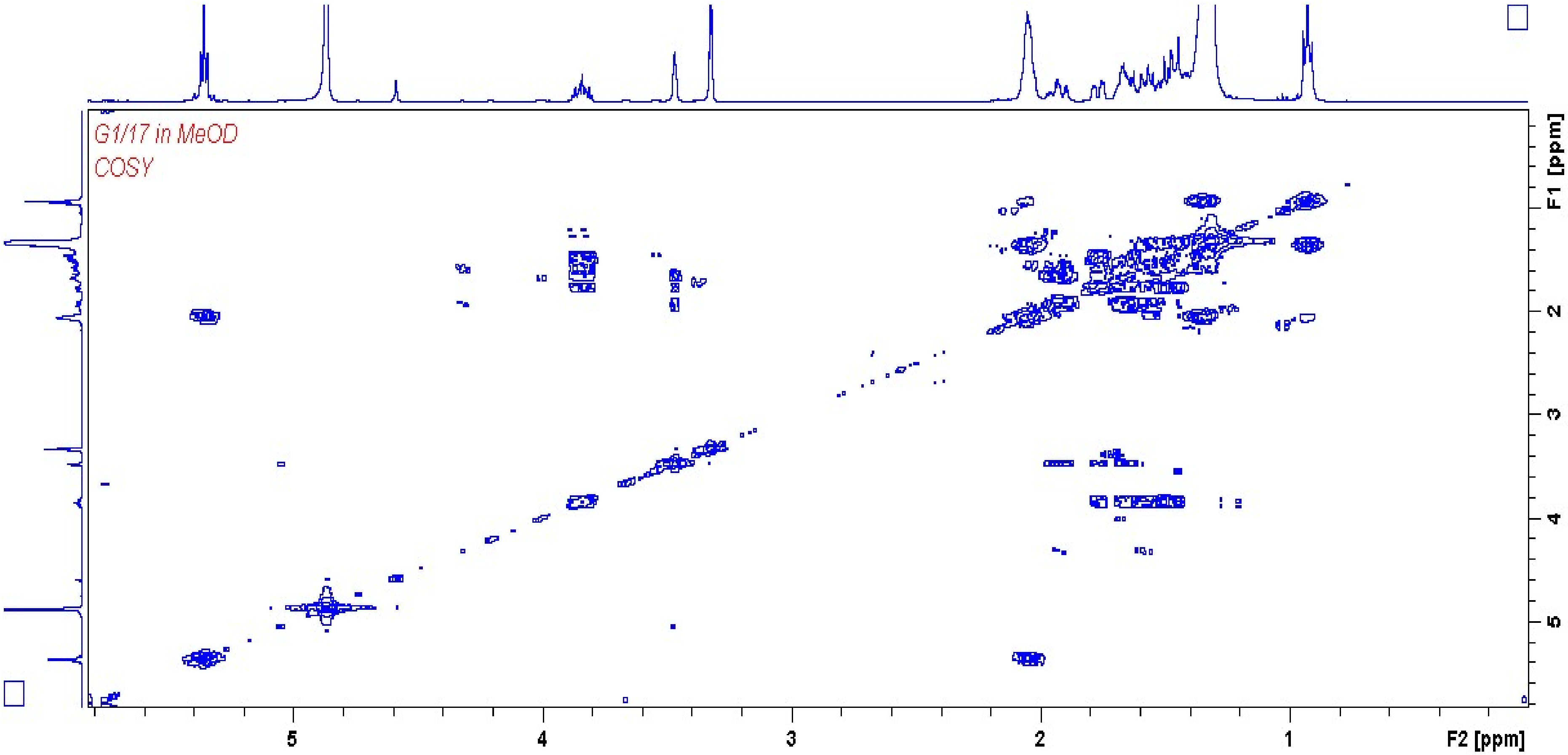

Figure S32. COSY spectrum for (1*R*,2*S*,4*R*)-1,2,4-trihydroxy-4-[heptadec-12′(*Z*)-enyl]cyclohexane (**4**)

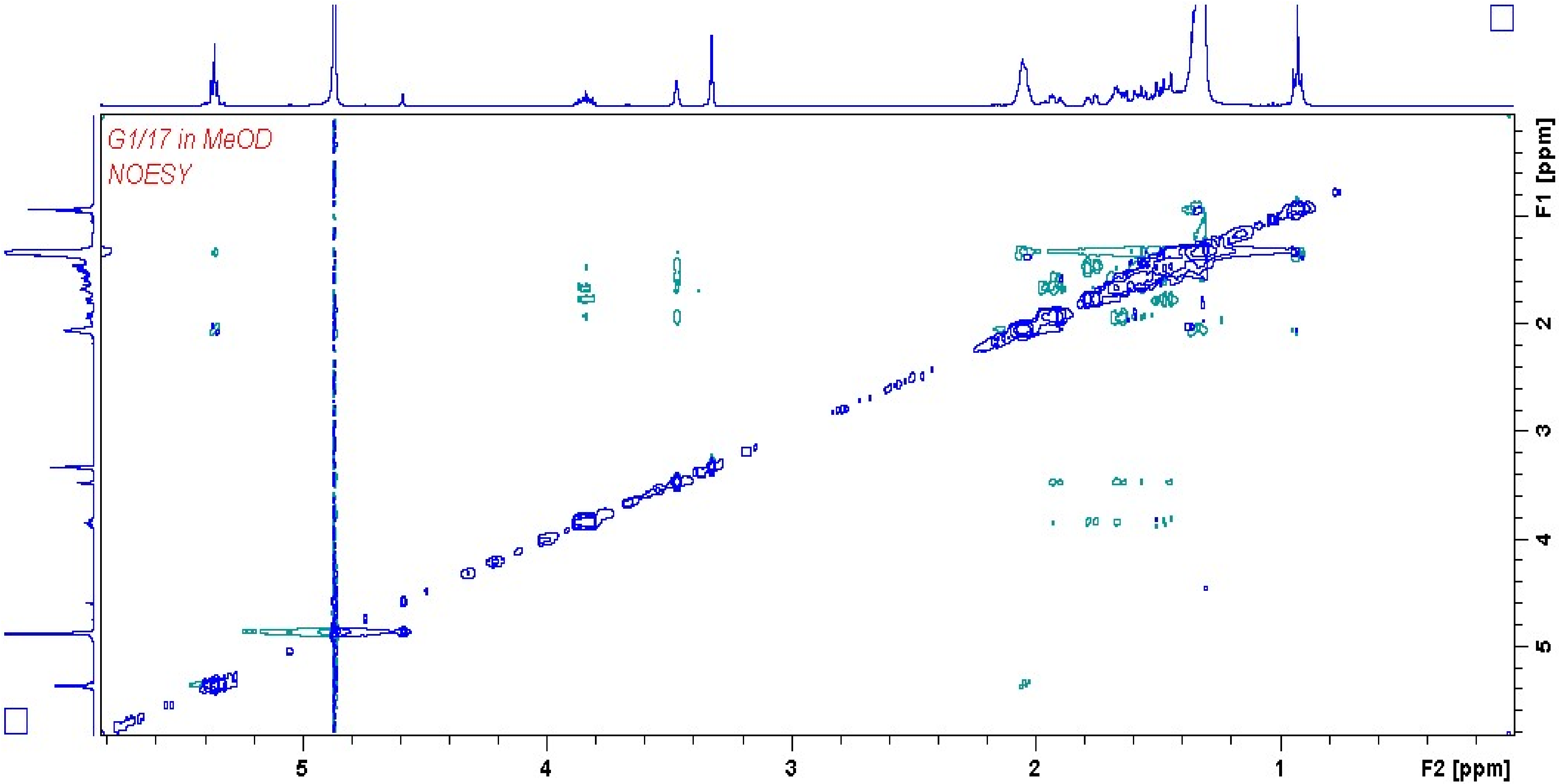

Figure S33. NOESY spectrum for (1*R*,2*S*,4*R*)-1,2,4-trihydroxy-4-[heptadec-12′(*Z*)-enyl]cyclohexane (**4**)

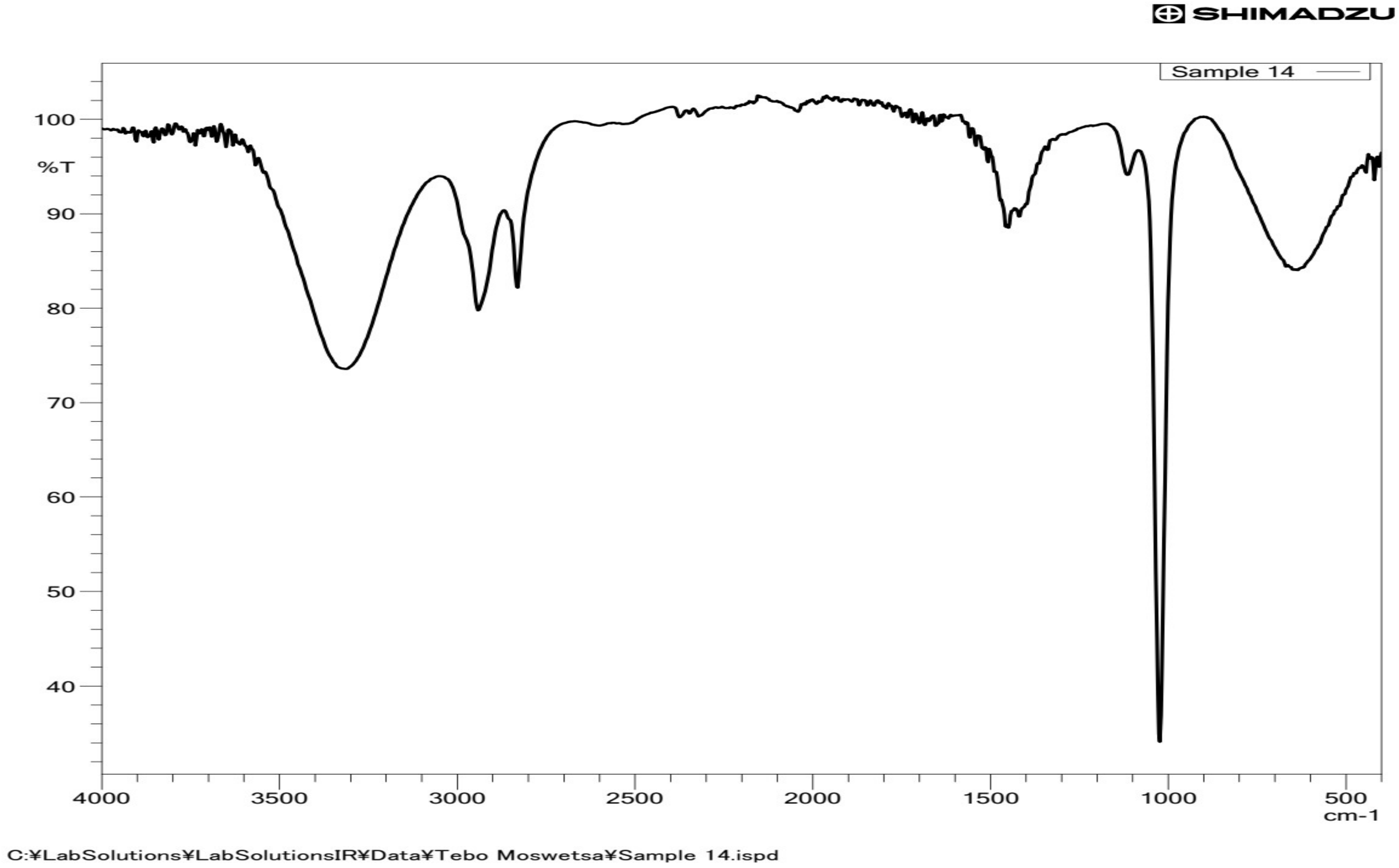

Figure S34. FTIR spectrum for (1*R*,2*S*,4*R*)-1,2,4-trihydroxy-4-[heptadec-12′(*Z*)-enyl]cyclohexane (**4**)

Elemental Composition Report Page 1

Single Mass Analysis
Tolerance = 10.0 mDa / DBE: min = -1.5, max = 100.0
Element prediction: Off
Number of isotope peaks used for i-FIT = 3

Monoisotopic Mass, Even Electron Ions
72 formula(e) evaluated with 1 results within limits (up to 10 best isotopic matches for each mass)
Elements Used:
C: 1-30 H: 1-100 O: 0-20

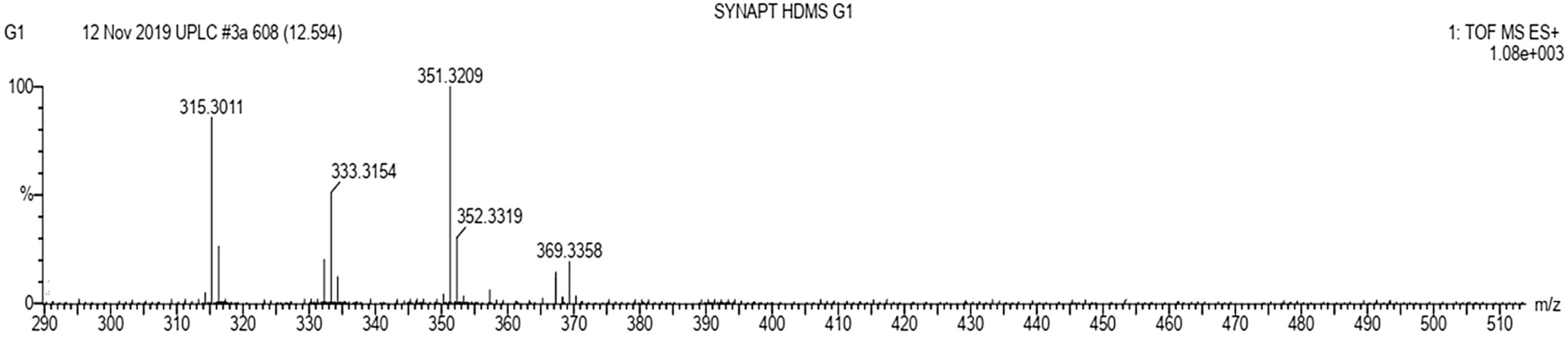

Minimum:				-1.5			
Maximum:		10.0	10.0	100.0			
Mass	Calc. Mass	mDa	PPM	DBE	i-FIT	i-FIT (Norm)	Formula
369.3358	369.3369	-1.1	-3.0	1.5	21.1	0.0	C23 H45 O3

Figure S35. HRMS for (1*R*,2*S*,4*R*)-1,2,4-trihydroxy-4-[heptadec-12′(*Z*)-enyl]cyclohexane (**4**)

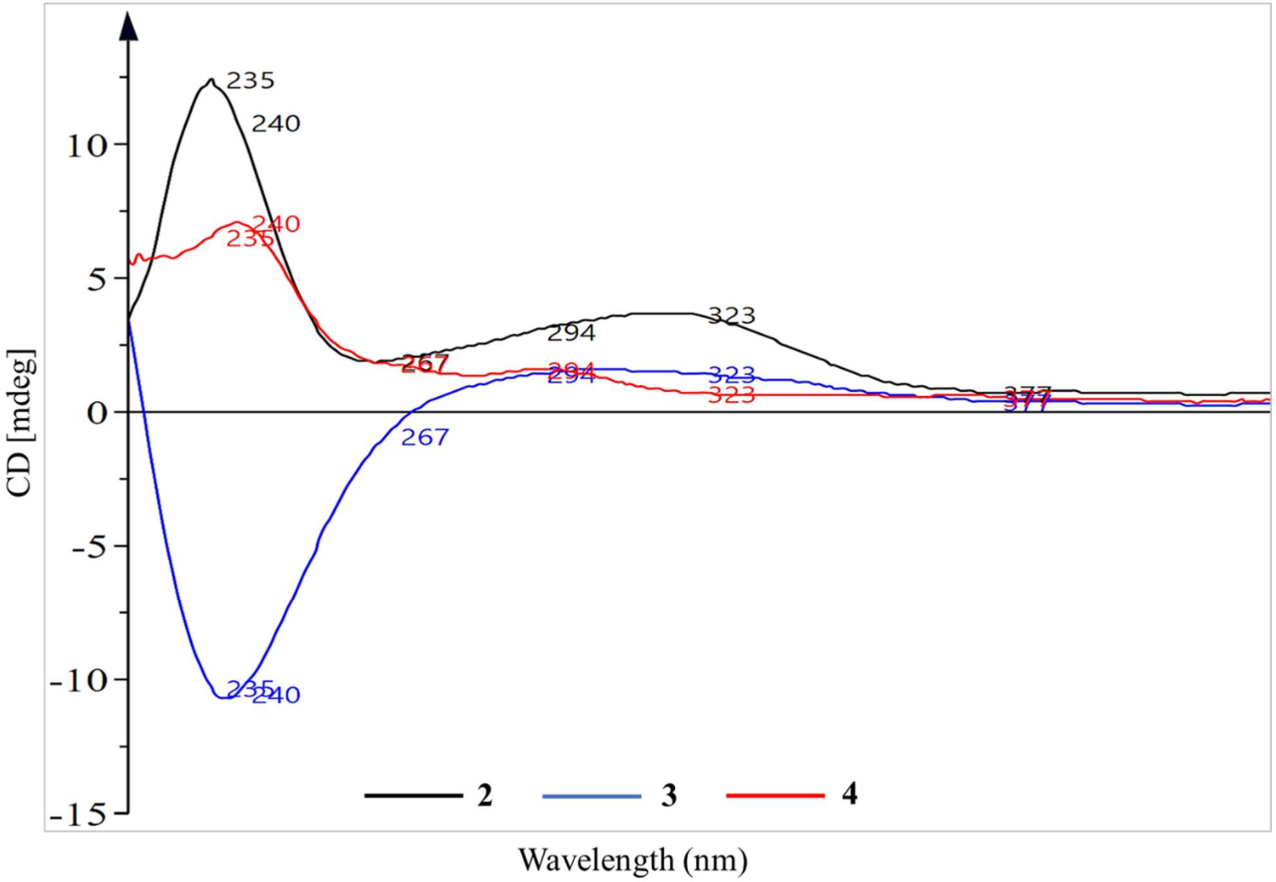

Figure S36. ECD spectra for compounds **2** – **4**.
